# DBP: Adaptive and Interpretable Factor Analysis for Single-cell RNA-seq Data with Deep Beta Processes

**DOI:** 10.1093/gpbjnl/qzaf117

**Published:** 2025-11-26

**Authors:** Runyan Liu, Shuofeng Hu, Guohua Dong, Tongtong Kan, Jinhui Shi, Jing Wang, Jiahao Zhou, Zhen He, Xiaomin Ying

**Affiliations:** Center for Computational Biology, Beijing Institute of Basic Medical Sciences, Beijing 100850, China; Center for Computational Biology, Beijing Institute of Basic Medical Sciences, Beijing 100850, China; Center for Computational Biology, Beijing Institute of Basic Medical Sciences, Beijing 100850, China; Center for Computational Biology, Beijing Institute of Basic Medical Sciences, Beijing 100850, China; Center for Computational Biology, Beijing Institute of Basic Medical Sciences, Beijing 100850, China; Center for Computational Biology, Beijing Institute of Basic Medical Sciences, Beijing 100850, China; Center for Computational Biology, Beijing Institute of Basic Medical Sciences, Beijing 100850, China; College of Electronics and Information Engineering, Shenzhen University, Shenzhen 518060, China; Center for Computational Biology, Beijing Institute of Basic Medical Sciences, Beijing 100850, China; Center for Computational Biology, Beijing Institute of Basic Medical Sciences, Beijing 100850, China

**Keywords:** Single-cell, Factor analysis, Self-adaption, Interpretability, Deep Beta Process

## Abstract

Factor analysis is a method that condenses multiple variables into a few latent factors. It can be used to extract the underlying sources of biological variation in high-dimensional data and distill them into interpretable gene programs. However, existing factorization methods lack adaptability in selecting the optimal number of factors and interpretability in capturing biological variation. To address these concerns, we propose Deep Beta Process (DBP), a deep probabilistic framework for adaptive and interpretable factor analysis of single-cell transcriptomic data. DBP achieves adaptive selection of factors through a stick-breaking Beta process and performs batch correction using an adversarial learning strategy. We validate the flexible factor extraction and robust batch correction capabilities of DBP on simulated datasets. We also demonstrate its superior performance in dimensionality reduction and biological interpretability while explaining biological variation from both cell and gene perspectives using factor and loading matrices. The application of DBP to a gastric adenocarcinoma dataset reveals malignant epithelial cell heterogeneity, providing valuable insights for investigating the molecular mechanisms of disease onset and progression. DBP is available at https://github.com/labomics/DBP and https://ngdc.cncb.ac.cn/biocode/tool/BT007954.

## Graphical abstract

**Figure:**
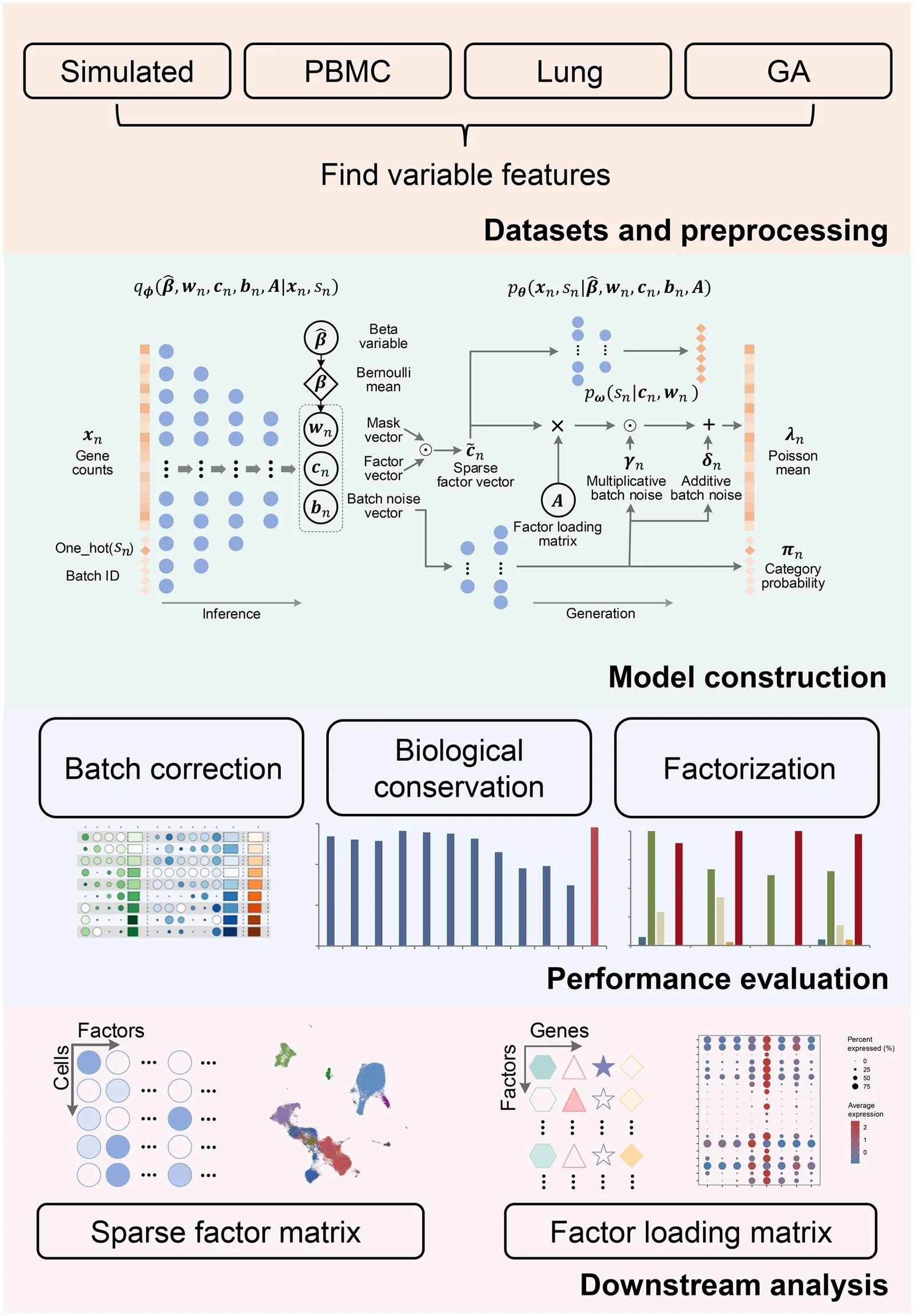

## Introduction

Single-cell RNA sequencing (scRNA-seq) [1] is a valuable tool for deciphering cell heterogeneity. A crucial challenge in scRNA-seq data analysis is to reveal inherent and interpretable gene programs (GPs) with biological variation. GPs comprise genes with coordinated expression and are responsible for complex cellular activities, which can be interpreted as cell types, cell functions, and biological processes.

Factorization serves as a core approach for mining candidate GPs. Various factorization methods have been proposed and widely applied for single-cell analysis [2–12]. However, these methods cannot efficiently capture the complexity of biological signals in single-cell data due to their linear nature. The low-dimensional representations of these methods inadequately capture the intrinsic structure in expression states and can only capture limited biological variation along linear axes within high-dimensional space. In contrast, deep learning-based methods, especially autoencoders (AEs), have been proposed due to their capabilities in handling large-scale, high-dimensional data and non-linear modeling, for instance, scAlign and MARS [13,14]. However, the interpretability of these methods is limited, as they require post hoc analyses to determine model parameters and distill biological signals [15,16].

Several methods that combine the variational autoencoder (VAE) [17] framework to achieve interpretability have been proposed. For example, LDVAE, scETM, VEGA, and siVAE [15,16,18,19] explain genomic features by leveraging the relationship between the neural network layers. However, these methods lack adaptability, as they require iterative subjective selections of the number of GPs, which is trivial and may often turn out to be unreasonable. Subjective determination may not only lead to the loss or duplication of GPs, but also hinders the flexible capture of the underlying factors reflecting biological variation. Moreover, some methods like LDVAE may still be influenced by batch noise, which can affect their biological interpretability. However, caution should be exercised when employing independent batch correction methods during data preprocessing, as they may inadvertently eliminate subtle biological signals [20,21]. Therefore, the interpretation of GPs continues to encounter challenges related to interpretability, adaptability, and robustness.

To address these limitations, we developed Deep Beta Process (DBP), a method for adaptive and interpretable factor analysis of single-cell transcriptomic data. By employing a stick-breaking Beta process, DBP generates a binary matrix as a mask, enabling both the adaptive selection of factors and cells for each factor, which offers two key advantages. First, it automatically determines the optimal number of factors based on the intricacy and meaning of the observed data. Second, it effectively selects key cells associated with each GP, thereby enhancing interpretability. To mitigate the impact of batch noise on GP interpretation, we incorporated a batch correction module that was jointly optimized with the factor analysis module. Benchmarking results on peripheral blood mononuclear cell (PBMC) and lung datasets [22,23] demonstrated that DBP can adaptively and robustly identify interpretable factors. Applying DBP to a gastric adenocarcinoma (GA) dataset [24] effectively delineated the heterogeneity of malignant epithelial cells and highlighted the genes associated with distinct subtypes of these cells.

## Method

### DBP modeling of single-cell transcriptomic data

Given a gene expression matrix $X=[x_{1};\;\ldots ;\;x_{N}]\in N^{N\times G}$ with N cells and G genes, let $s=[s_{1};\;\ldots ;\;s_{N}]\in \{1,2,\;\ldots ,\;{S\}}^{N\times 1}$ represent the corresponding batch IDs, where S denotes the number of batches. We define a factor matrix $C=[c_{1};\;\ldots ;\;c_{N}]\in R^{N\times \infty }$ and a batch noise matrix $\;B=[b_{1};\;\ldots ;\;b_{N}]\in R^{N\times M}$ as the latent variables for different cells, where M is the dimension of the batch noise. To disentangle the factors from the batch noise, we assume that C and B are mutually independent, and that the batch IDs s depend only on B.

To enable adaptive factor selection, we use a stick-breaking Beta process to generate a binary mask matrix $W=[w_{1};\;\ldots ;\;w_{N}]\in {\left\{0,1\right\}}^{N\times \infty }$ for C. Each row in W is drawn from distributions generated from the global latent variable $\hat{\beta }=[{\hat{\beta }}_{1},\ldots ,\;{\hat{\beta }}_{k},\ldots ]\in {\left[0,1\right]}^{\infty }$, where k denotes the index of the factor. This allows the prior sparsity to be adjusted in a data-driven manner, accommodating varying data complexities, and defines the cellular activity states with a limited number of factors. To formalize the gene weighting mechanism, we introduce a factor loading matrix $A=[a_{1};\;\ldots ;\;a_{k};\;\ldots \;]$, where $a_{k}$ denotes a specific GP and also acts as a global latent variable.

The generative process for the observed variables X and s is modeled by factorizing the joint distribution of all variables as follows (see File S1 for detailed derivation):


(1) $$\begin{array}{l} \begin{array}{l} p(X,\;s,\;\hat{\beta },\;W,\;C,\;B,\;A)\\ =p(\hat{\beta },\;A)p(X,\;s,\;W,C,\;B|\hat{\beta },\;A)\\ =p(\hat{\beta })p(A)p(W|\hat{\beta })p(C)p(B)p(s|B)p(X|W,\;C,\;B,\;A)\\ =\prod_{k=1}^{\infty }p({\hat{\beta }}_{k})p(a_{k})\prod_{n=1}^{N}p(w_{n}|\hat{\beta })p(c_{n})p(b_{n})p(s_{n}|b_{n})p(x_{n}|w_{n},c_{n},b_{n},A) \end{array} \end{array}$$

The corresponding probabilistic graphical model is depicted in Figure S1A. Specifically, the global variables $\hat{\beta }$ and A are generated first. For each k=1, 2, …, we have:


(2) $$\begin{array}{l} \begin{array}{l} p({\hat{\beta }}_{k})=\mathrm{Beta}(\alpha ,\;1)\\ p(a_{k})=\mathrm{Normal}(0,\;\sigma^{2}I) \end{array} \end{array}$$

Here, the prior $p({\hat{\beta }}_{k})$ is modeled as a Beta distribution with hyperparameters α and 1, while $p(a_{k})$ is an isotropic Gaussian distribution with variance $\sigma^{2}$. Conditioned on $\hat{\beta }$ and A, the local variables ***W***, ***C***, ***B***, ***s***, and X are generated. Specifically, for each n=1, … ,N:


(3) $$\begin{array}{l} \begin{array}{l} p(w_{n}|\hat{\beta })=\mathrm{Bernoulli}(\beta )\\ p(c_{n})=\mathrm{Normal}(0,\;I)\\ p(b_{n})=\mathrm{Normal}(0,\;I)\\ \pi_{n},\;\gamma_{n},\;\delta_{n}=\mathrm{NN}(b_{n};\;\theta )\\ p_{\theta }(s_{n}|b_{n})=\mathrm{Categorical}(\pi_{n})\\ \log\;\lambda_{n}=[(w_{n}⊙c_{n})A]⊙\gamma_{n}+\delta_{n}\\ p_{\theta }(x_{n}|w_{n},\;c_{n},\;b_{n},\;A)=\mathrm{Poisson}(\lambda_{n}) \end{array} \end{array}$$

Here, the prior $p(w_{n}|\hat{\beta })$ is modeled as a Bernoulli distribution with mean $\beta =[\beta_{1},\;\ldots ,\;\beta_{k},\;\ldots ]\in {\left[0,1\right]}^{\infty }$, where $\beta_{k}=\prod_{k^{\prime }=1}^{k}{\hat{\beta }}_{k^{\prime }}$. The priors $p(c_{n})$ and $p(b_{n})$ are standard Gaussian distributions. A neural network NN with learnable parameters θ is used to generate the probability vector $\pi_{n}\in \Delta^{S-1}$, the multiplicative batch noise $\gamma_{n}$, and the additive batch noise $\delta_{n}$. The likelihood $p_{\theta }(s_{n}|b_{n})$ is modeled as a Categorical distribution with mean $\pi_{n}$. Meanwhile, the likelihood $p(x_{n}|w_{n},\;c_{n},\;b_{n},\;A)$ is modeled as a Poisson distribution with mean $\lambda_{n}$. The logarithm of $\lambda_{n}$ is generated via a linear transformation involving ***w**_n_*, ***c**_n_*, ***A**_n_*, $\gamma_{n}$, and $\delta_{n}$, where ⊙ denotes the Hadamard product and we call ${\tilde{c}}_{n}=c_{n}⊙w_{n}$ as the sparse factor vector.

### Structured stochastic variational inference for DBP

Given an observed data matrix X and batch IDs s, our goal is to train the model parameters θ and infer the posterior distribution p(Z|X, s) over the latent variables $Z=\{\hat{\beta },\;W,\;C,\;B,\;A\}$. This can be achieved by employing stochastic gradient variational Bayes [25], which aims to maximize the expected evidence lower bound (ELBO) for the observed data (see File S2 for detailed derivation):


(4) $$\begin{array}{l} \mathrm{ELBO}(\theta ,\phi ;X,s)\\ ≜E_{q_{\phi }(Z|X,\;s)}[\log\;p_{\theta }\left(X,s|Z\right)]-\mathrm{KL}(q_{\phi }(Z|X,s)\|p(Z))\\ =\sum_{n=1}^{N}\begin{array}{l} \left[\begin{array}{l} E_{q_{\phi }(\overset{⁁}{\beta }|X,\;s)}E_{q_{\phi }(w_{n}|x_{n},\;s_{n},\;\overset{⁁}{\beta })}E_{q_{\phi }(c_{n}|x_{n},\;s_{n})}E_{q_{\phi }(b_{n}|x_{n},\;s_{n})}E_{q_{\phi }(A|X,\;s)}[\log\;p(x_{n}|w_{n},c_{n},b_{n},A)]\\ +E_{q_{\phi }(b_{n}|x_{n},\;s_{n})}[\log\;p_{\theta }(s_{n}|b_{n})]-E_{q_{\phi }(\overset{⁁}{\beta }|X,\;s)}[\mathrm{KL}(q_{\phi }(w_{n}|x_{n},s_{n},\;\overset{⁁}{\beta })\|p(w_{n}|\overset{⁁}{\beta }))]\\ -\mathrm{KL}(q_{\phi }(c_{n}|x_{n},s_{n})\|p(c_{n}))-\mathrm{KL}(q_{\phi }(b_{n}|x_{n},s_{n})\|p(b_{n})) \end{array}\right] \end{array}\\ -\sum_{k=1}^{\infty }\left[\mathrm{KL}(q_{\phi }{(\overset{⁁}{\beta }}_{k}|X,s)\|p{(\overset{⁁}{\beta }}_{k}))+\mathrm{KL}(q_{\phi }(a_{k}|X,s)\|p(a_{k}))\right] \end{array}$$

Here, $q_{\phi }(Z|X,s)$ is the variational approximation of the true posterior distribution p(Z|X,s), modeled by neural networks with parameters ϕ. The Kullback-Leibler divergence, denoted as KL(⋅‖⋅), quantifies the discrepancy between two distributions. To effectively capture the dependencies among the latent variables in the posterior $q_{\phi }(Z|X,s)$, we employ structured variational inference [26]. This involves factorizing $q_{\phi }(Z|X,s)$ into several components including $q_{\phi }({\hat{\beta }}_{k}|X,s)$, $q_{\phi }(w_{n}|x_{n},s_{n},\hat{\beta })$, $q_{\phi }(c_{n}|x_{n},s_{n})$, $q_{\phi }(b_{n}|x_{n},s_{n})$, and $q_{\phi }(a_{k}|X,s)$ and prioritizing their inference (see Figure S1B for the inference process). For $q_{\phi }({\hat{\beta }}_{k}|X,s)$ and $q_{\phi }(a_{k}|X,s)$, which are conditioned on the entire dataset, the distribution parameters are directly learned. These distributions can be written as $q_{\phi }({\hat{\beta }}_{k})$ and $q_{\phi }(a_{k})$, respectively. For $q_{\phi }(w_{n}|x_{n},s_{n},\hat{\beta })$, $q_{\phi }(c_{n}|x_{n},s_{n})$, and $q_{\phi }(b_{n}|x_{n},s_{n})$, which are conditioned on individual data points, the distribution parameters are generated by neural networks. Based on Equation 4, the ELBO for each individual data point ${\{x}_{n},s_{n}\}$ can be expressed as:


(5) $$\begin{array}{l} \begin{array}{l} \mathrm{ELBO}(\theta ,\phi ;x_{n},s_{n})\\ =E_{q_{\phi }(\hat{\beta })}E_{q_{\phi }(w_{n}|x_{n},\;s_{n},\;\hat{\beta })}E_{q_{\phi }(c_{n}|x_{n},\;s_{n})}E_{q_{\phi }(b_{n}|x_{n},\;s_{n})}E_{q_{\phi }(A)}[\log\;p(x_{n}|w_{n},c_{n},b_{n},A)]\\ +E_{q_{\phi }(b_{n}|x_{n},\;s_{n})}[\log\;p_{\theta }(s_{n}|b_{n})]-E_{q_{\phi }(\hat{\beta })}[\mathrm{KL}(q_{\phi }(w_{n}|x_{n},s_{n},\hat{\beta })\|p(w_{n}|\hat{\beta }))]\\ -\mathrm{KL}(q_{\phi }(c_{n}|x_{n},s_{n})\|p(c_{n}))-\mathrm{KL}(q_{\phi }(b_{n}|x_{n},s_{n})\|p(b_{n}))\\ -\frac{1}{N}\sum_{k=1}^{\infty }\left[\mathrm{KL}(q_{\phi }{(\hat{\beta }}_{k})\|p{(\hat{\beta }}_{k}))+\mathrm{KL}(q_{\phi }(a_{k})\|p(a_{k}))\right] \end{array} \end{array}$$

To maximize Equation 5, we can minimize the loss function as follows:


(6) $$\begin{array}{l} L^{\mathrm{ELBO}}(\theta ,\;\phi ;\;x_{n},s_{n})≜-\mathrm{ELBO}(\theta ,\phi ;x_{n},s_{n}) \end{array}$$

### Batch correction via adversarial learning

Inspired by the studies of Cao et al. [27] and Shree et al. [28], which employ adversarial training strategies for batch correction, we first use a discriminator to predict the batch ID of each cell based on the sparse factor ${\tilde{c}}_{n}$. The training process involves minimizing the cross-entropy loss between the predicted and actual batch IDs:


(7) $$\begin{array}{l} L^{D}(\omega ;x_{n},s_{n},\phi )≜-E_{q_{\phi }({c_{n},\;w}_{n}|x_{n},\;s_{n})}\log\;p_{\omega }(s_{n}|c_{n},w_{n}) \end{array}$$

where ω represents the parameters of the discriminator. Subsequently, we train the encoder in the opposite direction to fool the discriminator:


(8) $$\begin{array}{l} L^{A}(\phi ;x_{n},s_{n},\omega )≜E_{q_{\phi }({c_{n},w}_{n}|x_{n},s_{n})}\log\;p_{\omega }(s_{n}|c_{n},w_{n}) \end{array}$$

By optimizing both the encoder and the discriminator, the final discriminator becomes incapable of distinguishing the batch IDs of cells from the sparse factors generated by the encoder, thereby achieving effective batch correction.

### Training DBP

Combining Equations 6 and 8, the overall training loss for each cell is given by:


(9) $$\begin{array}{l} L(\theta ,\phi ;x_{n},s_{n},\omega )=L^{\mathrm{ELBO}}(\theta ,\phi ;x_{n},s_{n})+L^{A}(\phi ;x_{n},s_{n},\omega ) \end{array}$$

We alternately minimize Equations 7 and 9 using stochastic gradient descent (SGD), which forms the DBP training algorithm (**Algorithm 1**).

Algorithm 1 The DBP training algorithm

**Input:** A single-cell RNA-seq dataset ${\left\{x_{n},\;s_{n}\right\}}_{n=1}^{N}$

**Output:** Decoder parameters θ, encoder parameters ϕ, and discriminator parameters ω

1: Initialize {θ, ϕ, ω} randomly

2: **for** Iteration t=1, 2, …, T  **do** 

3: Sample a minibatch ${\left\{x_{n},\;s_{n}\right\}}_{n\in N_{t}}$ from the dataset, where $N_{t}\subset \{1,2,\;\ldots ,\;N\}$

4: **for** Step d=1,2, …, D  **do** 

5:   Freeze ϕ and update ω by minimizing the loss $\frac{1}{\left|N_{t}\right|}\sum_{n\in N_{t}}L^{D}(\omega ;\;x_{n},\;s_{n},\;\phi )$              **▷** See Equation 7

6: **end for** 

7: Freeze ω and update {θ, ϕ} by minimizing the loss $\frac{1}{\left|N_{t}\right|}\sum_{n\in N_{t}}L(\theta ,\;\phi ;\;x_{n},\;s_{n},\;\omega )$            **▷** See Equation 9

8: **end for** 

### Sparse factor selection

The accurate determination of the number of sparse factors is pivotal in factor analysis for deciphering GPs. To identify interpretable sparse factors from the sparse factor matrix $\tilde{C}=C⊙W$ (with a truncation level of 100) [29,30]. Here, $w_{n,k}$ equals 1 if factor *k* is active for the n-th cell and 0 otherwise. The truncation level is introduced to ensure computational feasibility by approximating the infinite-dimensional process with a finite-dimensional representation [31,32].

Subsequently, we calculated the ratio between the sum of sparse factor values within each factor and the total sum across all factors’ sparse factor values, referred to as the cumulative contribution rate (CCR). The selection threshold was set at CCR(*k*) > 99%, with the smallest k satisfying this condition determining the retained sparse factors. Consequently, factors that are active in only a negligible subset of cells are discarded, enabling the adaptive determination of the number of latent factors.


(10) $$\begin{array}{l} \mathrm{CCR}(k)=\frac{\sum_{k^{\prime }=1}^{k}\sum_{n=1}^{N}{\tilde{c}}_{n,k^{\prime }}}{\sum_{k^{\prime }=1}^{\infty }\sum_{n=1}^{N}{\tilde{c}}_{n,k^{\prime }}} \end{array}$$

where ${\tilde{c}}_{n,k}$ represents the k-th sparse factor of cell n.

### Definition of sparse factors

The sparse factors obtained through DBP adaptation represent distinct GPs, while the sparse factor matrix and the factor loading matrix capture the activity levels of cells and genes associated with these GPs. In the sparse factor matrix, each cell value of a factor is distributed along a coordinate axis centered at 0. Different directions represent opposite phenotypes, and the magnitude of the absolute value indicates the level of activity. We select the most biologically interpretable direction as the representative GP for each sparse factor. In relation to the factor loading matrix, we identify genes that have the same direction as the sparse factors as top genes, while those that display opposing directions are referred to as tail genes.

### Gene set enrichment analysis

To investigate the relationship between the sparse factors and known GPs, we need to analyze the functional significance of the top genes. However, determining the appropriate threshold for selecting the top genes in factor analysis can be challenging. To overcome this, we employed the ‘prerank’ module from the GSEApy package [33] to annotate the gene sets associated with the factor loadings. Here, we used the sorted values of $a_{k}$ along with their corresponding gene names as the pre-ranked gene list dataset, referred to as the correlation value file (.rnk). Additionally, we selected pathway C5 (ontology gene sets) from the MSigDB database to serve as the gene set file (.gmt).

### Evaluation metrics

#### Dimensionality reduction metrics

To quantitatively evaluate the performance of DBP and state-of-the-art methods on dimensionality reduction, we employ the single-cell integration benchmarking (scIB) tool [34] to calculate batch correction and biological conservation metrics based on $\tilde{C}$. The batch correction metrics include k-nearest-neighbor graph connectivity (graph connectivity), average silhouette width across batches [batch average silhouette width (ASW)], k-nearest-neighbor batch effect test (kBET), and graph integration local inverse Simpson’s index (graph iLISI). The biological conservation metrics consist of average silhouette width across cell types (cell type ASW), isolated label silhouette, isolated label F1, normalized mutual information (NMI), Adjusted Rand Index (ARI), and graph cell-type local inverse Simpson’s index (graph cLISI). Following Luecken et al. [34], the overall score is calculated by combining the average of batch correction metrics, weighted at 0.4, with the average of biological conservation metrics, which is weighted at 0.6.

#### Factorization metrics

In general, greater sparsity implies a reduced requirement for cells to explain most variables, thus indicating increased interpretability. To evaluate sparsity, we consider three commonly employed metrics: L2/L1 ratio (η), Kurtosis (κ), and zero element ratio (ZER; ρ). These metrics are computed as follows.

##### L2/L1

The L2/L1 metric computes the ratio between the L2 norm and the L1 norm. The corresponding score is then calculated as follows:


(11) $$\begin{array}{c} \eta =\frac{1}{\hat{K}}\sum_{k=1}^{\hat{K}}\frac{\sqrt{\sum_{n=1}^{N}{\tilde{c}}_{n,k}^{2}}}{\sum_{n=1}^{N}\left|{\tilde{c}}_{n,k}\right|} \end{array}$$

Here, $\hat{K}$ is the learned number of factors.

##### Kurtosis

Kurtosis is the ratio of the fourth central moment to the square of the variance. To calculate its value, we utilize the scipy.stats.kurtosis function from the SciPy package [35]. The Kurtosis score is then calculated as follows:


(12) $$\begin{array}{c} \kappa =\frac{1}{\hat{K}}\sum_{k=1}^{\hat{K}}\frac{\frac{1}{N}{\sum_{n=1}^{N}\left({\tilde{c}}_{n,k}-\mu_{k}^{\tilde{c}}\right)}^{4}}{{\left(\frac{1}{N}\sum_{n=1}^{N}{\left({\tilde{c}}_{n,k}-\mu_{k}^{\tilde{c}}\right)}^{2}\right)}^{2}} \end{array}$$

Here, $\mu_{k}^{\tilde{c}}$ represents the mean value of the k-th sparse factor.

##### ZER

ZER denotes the ratio of zero elements in a vector and is calculated as follows:


(13) $$\begin{array}{c} \rho =\frac{1}{\hat{K}N}\sum_{k=1}^{\hat{K}}\sum_{n=1}^{N}1({\tilde{c}}_{n,k}=0) \end{array}$$

Here, 1(⋅) is the 0–1 indicator function.

##### F statistic

The F statistic is used to evaluate the difference between key and irrelevant cells in the simulated dataset and is calculated using the scipy.stats.f_oneway function in the SciPy package.

### Datasets and preprocessing

#### Simulated data

Given that DBP is capable of batch correction while DBP_adap and DBP_naive are not, we utilized the SCSIM and Splatter packages to generate simulated single-batch and multi-batch datasets, respectively. The simulated single-batch dataset consists of 50,000 cells and 20,000 genes. To ensure diversity, we included 11 GPs, which comprise 10 cell type-specific GPs (GP1–10) and 1 shared GP between Cell Type 1 and Cell Type 2 (GP11). Detailed parameter settings are listed in Table S1.

The simulated multi-batch dataset comprises 50,000 cells and 10,000 genes. During the data generation process, we incorporated various challenges for batch correction. These include varying the number of cells per batch from 1800 to 3000 and adjusting the proportion of cell numbers and counts for different cell types within the same batch, ranging from 0% to 35% and from 28% to 45%, respectively. Detailed parameter settings are listed in Table S2.

#### PBMC data

The PBMC dataset was obtained from the Gene Expression Omnibus (GEO: GSE164378) [36]. We selected the transcriptomic data from eight healthy samples for our analysis.

#### Lung data

The lung dataset was obtained from the Human Cell Atlas Data Coordination Platform (https://www.tissuestabilitycellatlas.org/). This dataset consists of transcriptomic data from five healthy human lung samples.

#### GA data

The GA dataset was obtained from the Genome Sequence Archive for Human (GSA-Human: HRA000051) [37]. This dataset was derived from transcriptomic data of nine tumor samples and three non-tumor samples.

#### Data preprocessing

We utilized the R package Seurat for dataset preprocessing. Initially, a Seurat object was created using the CreateSeuratObject function, and low-frequency genes were filtered out. Subsequently, 4000 highly variable genes (HVGs) were selected for each batch using the FindVariableFeatures function. The HVGs then underwent integrated feature selection and ranking utilizing the SelectIntegrationFeatures function, resulting in the top 4000 highest-ranking genes across all batches.

### Implementation of DBP

We implemented the architecture of DBP using PyTorch. The encoder hidden layer size was set to 2000–1000, the number of factors was truncated to 100, and the batch noise dimension was set to 2. To construct each hidden layer, we utilized four functions: Linear, LayerNorm, Mish, and Dropout. We optimized the model using AdamW [38] as the optimizer, with a learning rate of $10^{-4}$ and a minibatch size of 256. The model was trained for a maximum of 2000 epochs, utilizing early stopping to terminate training. A dropout rate of 0.2 was applied to all hidden layers. All hyperparameters used for DBP training are detailed in Table S3.

### Implementation of methodological comparison

We conducted a comparison between DBP and eight reliable methods for batch correction, biological conservation, and biological interpretability performance. The comparison methods were divided into two categories: leading batch correction methods and interpretable factorization methods. The former group included Harmony (v0.1.1) [39], Seurat-CCA (v4.3.0) [40], Seurat-RPCA (v4.3.0) [40], and Scanorama (v1.7.2) [41], while the latter consisted of LIGER (v1.0.0) [6], MOFA+ (v1.4.0) [11], LDVAE [18], and scETM (v0.5.0).

#### Harmony

The R package Harmony is a method designed for batch correction. Initially, we used the NormalizeData, ScaleData, and RunPCA functions of the Seurat package to perform dimensionality reduction, and then applied the RunHarmony function of the Harmony package to remove batch effects.

#### LDVAE

The Python package LDVAE is an interpretable factorization method. We utilized the model.LinearSCVI function and the model.get_latent_representation function from the scvi-tools (v1.0.0) package to perform dimensionality reduction and GP extraction.

#### LIGER

The R package LIGER is a method for factorization and batch correction. We used the RunOptimizeALS and RunQuantileNorm functions of the rliger package to extract GPs (simultaneous dimensionality reduction) and remove batch effects.

#### MOFA+

The R package MOFA+ is a powerful factorization method. It applies factor pruning based on a user-specified minimum variance explained threshold during the training phase. We used the run_mofa function of the MOFA2 package to extract GPs (simultaneous dimensionality reduction). Additionally, the get_factors and get_weights functions were employed to obtain the factor matrix and factor loading matrix, respectively.

#### Scanorama

The Python package Scanorama (v1.7.2) is a method for batch correction. We used the integrate function of the Scanorama package for dimensionality reduction and batch effect removal.

#### scETM

The Python package scETM is a factorization method. We used the UnsupervisedTrainer function to extract GPs and the get_cell_embeddings_and_nll function to remove batch effects.

#### Seurat_CCA

Canonical correlation analysis (CCA) is an algorithm for batch correction in the R package Seurat. We first used the FindIntegrationAnchors function (reduction = ‘cca’) and the IntegrateData function to remove batch effects, and then used the RunPCA function to perform dimensionality reduction.

#### Seurat_RPCA

Seurat’s reciprocal principal component analysis (RPCA) is an algorithm designed for batch correction. In addition to setting reduction = ‘rpca’ in the FindIntegrationAnchors function, Seurat_RPCA uses the same strategy as Seurat_CCA.

## Results

### The DBP model

DBP is a deep generative model specifically designed for adaptive and interpretable factor analysis of scRNA-seq data. DBP assumes that the high-dimensional gene expression matrix is generated through the interaction of two low-dimensional matrices: a sparse factor matrix and a factor loading matrix. To enable adaptive selection of factors, DBP employs a Bayesian non-parametric approach to generate a mask matrix with an infinite number of columns, allowing for flexible and sparse selection of latent variable dimensions and cells (**Figure 1**A). The input of DBP consists of a gene-by-cell matrix and a vector of batch IDs. The output includes the factor matrix, mask matrix, and factor loading matrix. By taking the Hadamard product of the factor matrix and mask matrix, we obtain the sparse factor matrix which reveals factor-to-cell relationships and can be leveraged for various downstream biological analyses such as visualization, clustering, and cell type annotation. Furthermore, the factor loading matrix captures factor-to-gene relationships that can be utilized for gene set enrichment analysis (GSEA), biomarker identification, and gene set discovery.

**Figure 1:**
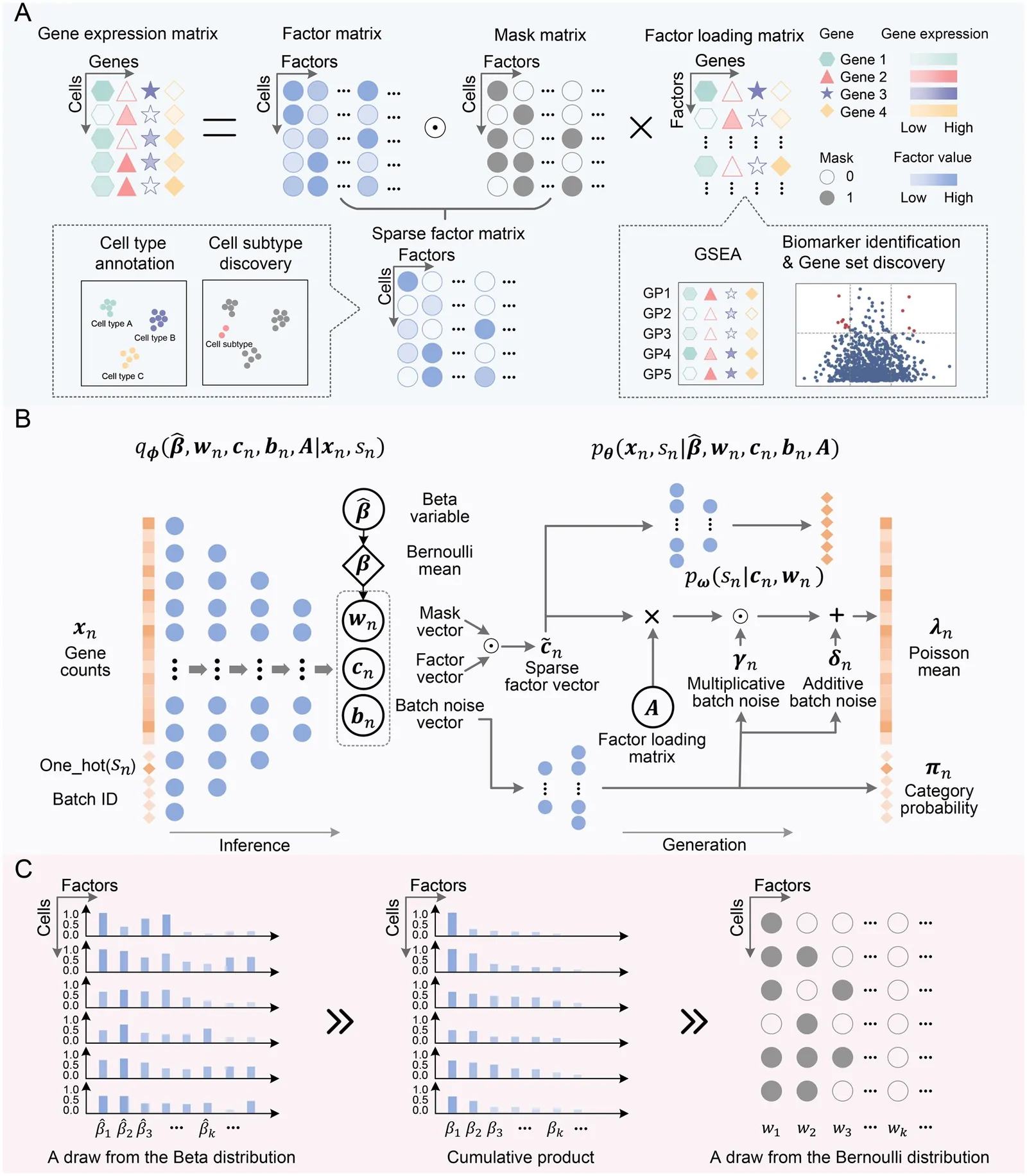
Overview of the DBP framework **A**. Adaptive and interpretable factor analysis. DBP assumes that the gene expression matrix is obtained by multiplying the sparse factor matrix and the factor loading matrix, where the sparse factor matrix is the Hadamard product of the factor matrix and the mask matrix. **B**. DBP model framework. DBP leverages a variational autoencoder for model inference and generation. A stick-breaking Beta process is used for adaptive factorization, and a discriminator is employed for batch correction. **C**. Stick-breaking Beta process. The process starts with sampling random numbers from a Beta distribution. These numbers are then cumulatively multiplied and integrated with the gene expression matrix data to derive probabilities for a Bernoulli distribution. Ultimately, binary matrices are sampled from this Bernoulli distribution. DBP, Deep Beta Process; GSEA, gene set enrichment analysis; GP, gene program.

DBP is constructed based on the architecture of a VAE to facilitate adaptive factor analysis and batch correction. The inference network estimates latent variables from the input data, while the generative network produces observed data using these latent variables. Adversarial learning strategies are employed to disentangle the sparse factor vector and batch noise vector, thus enabling effective batch correction (Figure 1B). Moreover, the stick-breaking Beta process approach is applied to initially sample an unbounded collection of probabilities within the unit interval from the Beta process, which are subsequently transformed into binary-valued values using a Bernoulli process for adaptive selection of both factors and cells [42] (Figure 1C).

### DBP achieves adaptive factor analysis and batch correction on simulated datasets

To demonstrate the functionalities of adaptive factor analysis and batch correction of DBP, we conducted an ablation study by using DBP and its two variants: DBP_adap and DBP_naive. The DBP_adap variant excludes the batch correction module from DBP (Figure S2A), and the DBP_naive variant eliminates both the batch correction module and the stick-breaking Beta process module (Figure S2B). To demonstrate the functionality of the modules, we used the single-cell next-generation sequencing simulator (SCSIM) [43] to construct a simulated single-batch dataset and Splatter [44] to construct a simulated multi-batch dataset (see Method).

We initially evaluated the stick-breaking Beta process module by comparing DBP_naive with DBP_adap on the simulated single-batch dataset. For DBP_naive, we employed various *K* values to generate multiple factor matrices and subsequently selected the one with the highest degree of biological conservation as the outcome, where *K* is the set number of factors. For DBP_adap, we obtained a data-driven sparse factor matrix comprising 16 factors. Uniform Manifold Approximation and Projection (UMAP) [45] visualization revealed that, compared to DBP_naive, DBP_adap effectively captured sparse factors representing the predefined 11 GPs (**Figure 2**A and B). scIB and F statistic benchmarking showed that in terms of biological conservation and interpretability, DBP_adap outperformed multiple experiments of DBP_naive with best results obtained at *K* = 12 (Figure 2C and D).

**Figure 2:**
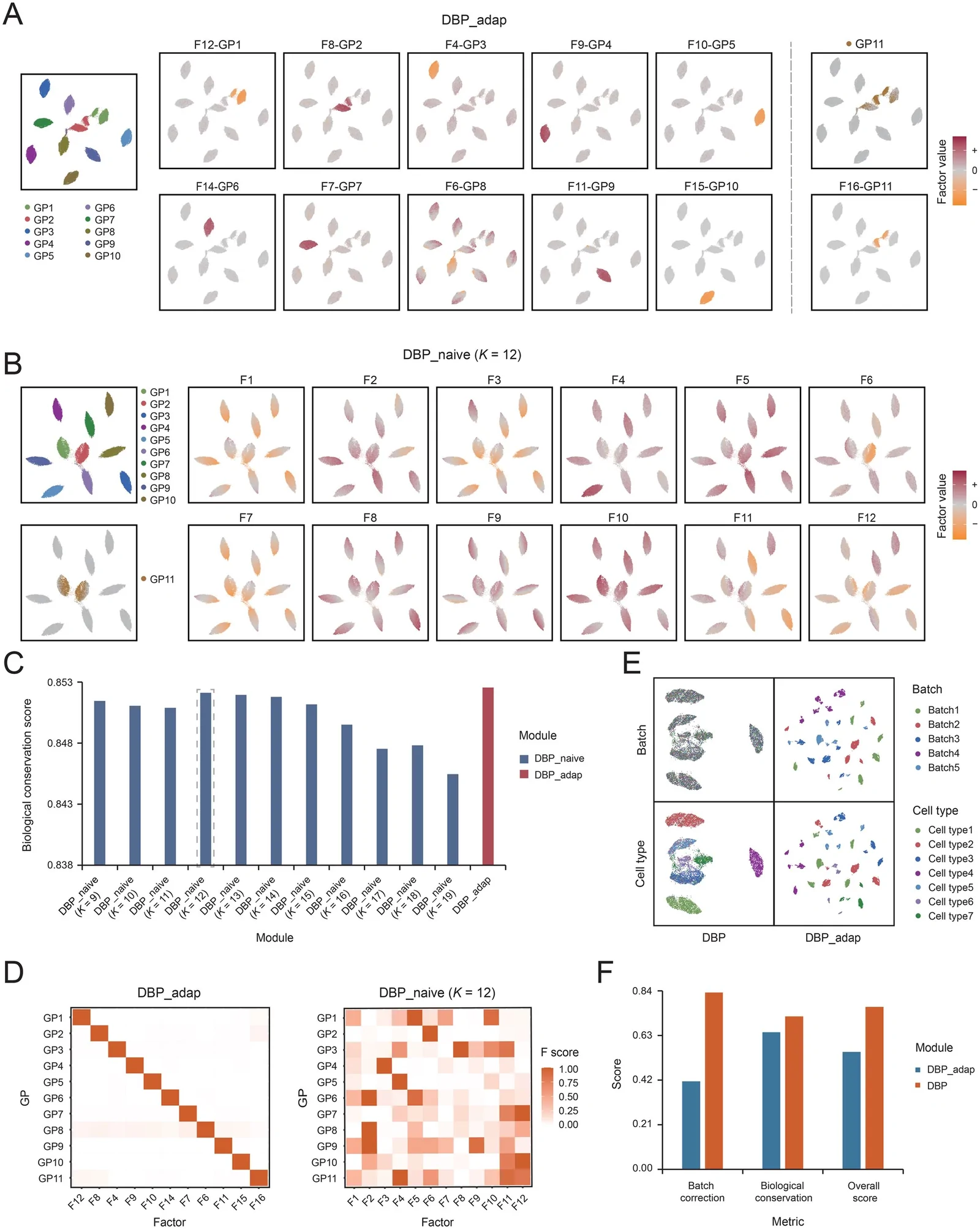
Ablation experiment results of DBP **A**. UMAP visualization of actual GPs and sparse factor vector values obtained by DBP_adap in the single-batch dataset. **B**. UMAP visualization of actual GPs and factor vector values obtained by DBP_naive (*K* = 12) in the single-batch dataset. The left panel represents the actual GPs, while the right panel displays all 12 factor vector values. **C**. Benchmarking the biological conservation performance of DBP_naive and DBP_adap on the single-batch dataset. **D**. F statistic scores comparing the performance of DBP_adap and DBP_naive (*K* = 12) on biological interpretability in the single-batch dataset. **E**. UMAP visualization of the sparse factor matrices obtained by DBP and DBP_adap in the multi-batch dataset. The top row is colored by batch, and the bottom row is colored by cell type. **F**. Benchmarking the dimensionality reduction performance of DBP_adap and DBP on the multi-batch dataset. UMAP, Uniform Manifold Approximation and Projection.

Subsequently, we evaluated the batch correction module by comparing DBP_adap with DBP on a simulated multi-batch dataset. The UMAP visualization and scIB benchmarking indicated that, compared to DBP_adap, DBP successfully preserved biological variation while achieving effective batch correction (Figure 2E and F). These results indicate that incorporating both the stick-breaking Beta process module and the batch correction module makes DBP a flexible and robust approach for adaptive factor analysis.

### Interpretable DBP exhibits superior performance in dimensionality reduction

Maintaining dimensionality reduction performance is crucial and presents challenges for interpretable factorization methods. To evaluate the performance of DBP in dimensionality reduction, we conducted both qualitative and quantitative comparisons with leading batch correction and interpretable factorization methods. This evaluation was performed using two publicly available single-cell transcriptomic datasets: the PBMC dataset, which has cell type-level labels, and the lung dataset, which has subtype-level labels (see Method). For the low-dimensional representations of the PBMC and lung datasets, DBP adaptively determined 38 and 50 dimensions, respectively, while MOFA+ automatically pruned these to 31 and 43 dimensions. To preserve spatial structure and GP coverage, we fixed the dimensionality of the other methods at 50 (**Figure 3**). Additionally, to ensure a rigorous comparison, we included configurations matching DBP’s adaptive dimensions to eliminate potential interference caused by differences in dimensionality (Figure S3).

**Figure 3:**
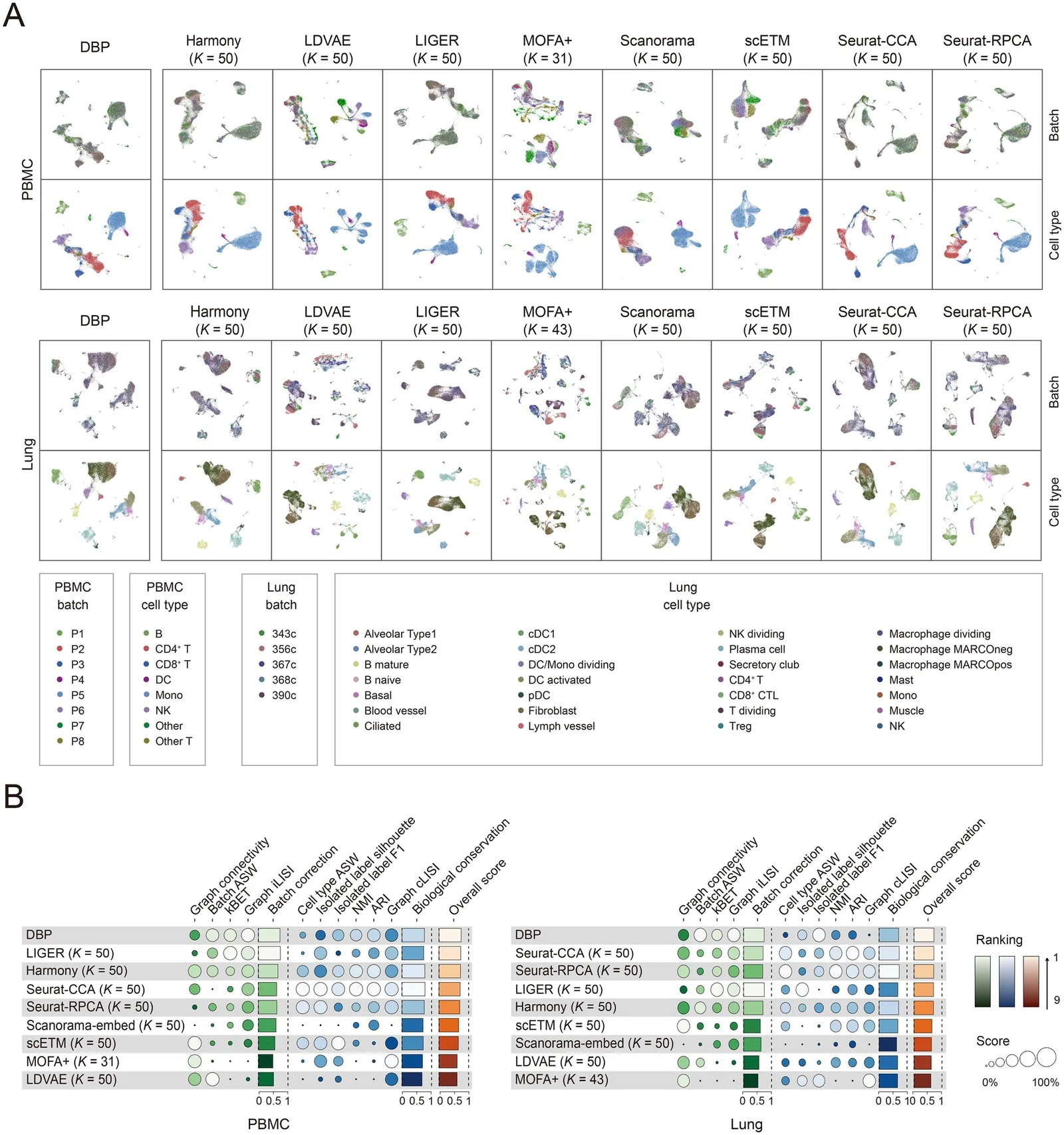
Evaluation of DBP performance on dimensionality reduction **A**. UMAP visualization of the sparse factor matrices obtained by DBP and the cell embeddings obtained by eight other methods in the PBMC and lung datasets. **B**. Benchmarking the dimensionality reduction performance of DBP and other methods on the PBMC and lung datasets. PBMC, peripheral blood mononuclear cell; DC, dendritic cell; Mono, monocyte; NK, natural killer; cDC, conventional dendritic cell; pDC, plasmacytoid dendritic cell; CTL, cytotoxic T lymphocyte; Treg, regulatory T; ASW, average silhouette width; kBET, k-nearest-neighbor batch effect test; iLISI, integration local inverse Simpson’s index; NMI, normalized mutual information; ARI, Adjusted Rand Index; cLISI, cell-type local inverse Simpson’s index.

In the qualitative evaluation, UMAP visualizations showed that DBP aligned cells of the same cell type across different batches while preserving their grouping and separation (Figure 3A, Figure S3A). However, other methods exhibited slightly inferior performance. For example, MOFA+, LDVAE, and scETM failed to effectively integrate the same cell types across batches, highlighting their limited batch-effect correction capabilities. Scanorama partially merged CD4^+^ and CD8^+^ T cells, indicating an overcorrection of batch effects and an inability to capture nuanced cell type differences.

In the quantitative evaluation, the scIB benchmarking results consistently aligned with the UMAP visualization results, demonstrating that methods that achieve superior low-dimensional representation also exhibit higher overall scores (Figure 3B, Figure S3B). In addition, compared to methods that tend to remove batch effects rather than preserve biological variation, leading to overcorrection, DBP exhibits stable dimensionality reduction capabilities. Furthermore, benchmarking of DBP’s performance under different hyperparameter settings, as well as its computational costs compared to other methods, indicates that DBP is a robust and efficient tool for adaptive and interpretable factor analysis of single-cell transcriptomic data (Figure S3C and D).

### DBP enables the definition of sparse factors with enhanced interpretability

The increasing diversity and quantity of scRNA-seq datasets require more interpretable low-dimensional representations. Most existing methods often produce difficult-to-interpret outcomes due to their lack of non-linearity, robustness, and flexibility. DBP is designed to adaptively learn interpretable sparse factors. To demonstrate the biological interpretability performance of DBP, we compared it with state-of-the-art interpretable factorization methods (see Method). To facilitate benchmarking, three interpretability evaluation metrics standardized the scales of diverse methods to fall within a range of 0 to 1 (see Method).

In the qualitative evaluation, we utilized a polar bubble chart (PBC) to visualize the distribution of normalized mean sparse factor values for each cell type within each sparse factor. The PBC visualization results showed that DBP more efficiently and effectively identifies interpretable GPs. For example, DBP extracts a greater number of GPs representing cell types (such as F17, F19, F24, F27, and F28 in the PBMC dataset) while masking irrelevant factor values as 0 (**Figure 4**A, Figure S4A). Similarly, UMAP visualization of sparse factor values corresponding to the known cell types indicated that DBP highlights key cells more prominently (Figure 4B, Figure S4B). The benchmarking results demonstrated that DBP has superior performance in interpretability (Figure 4C). These results demonstrate the ability of DBP to adaptively select key cells related to GPs.

**Figure 4:**
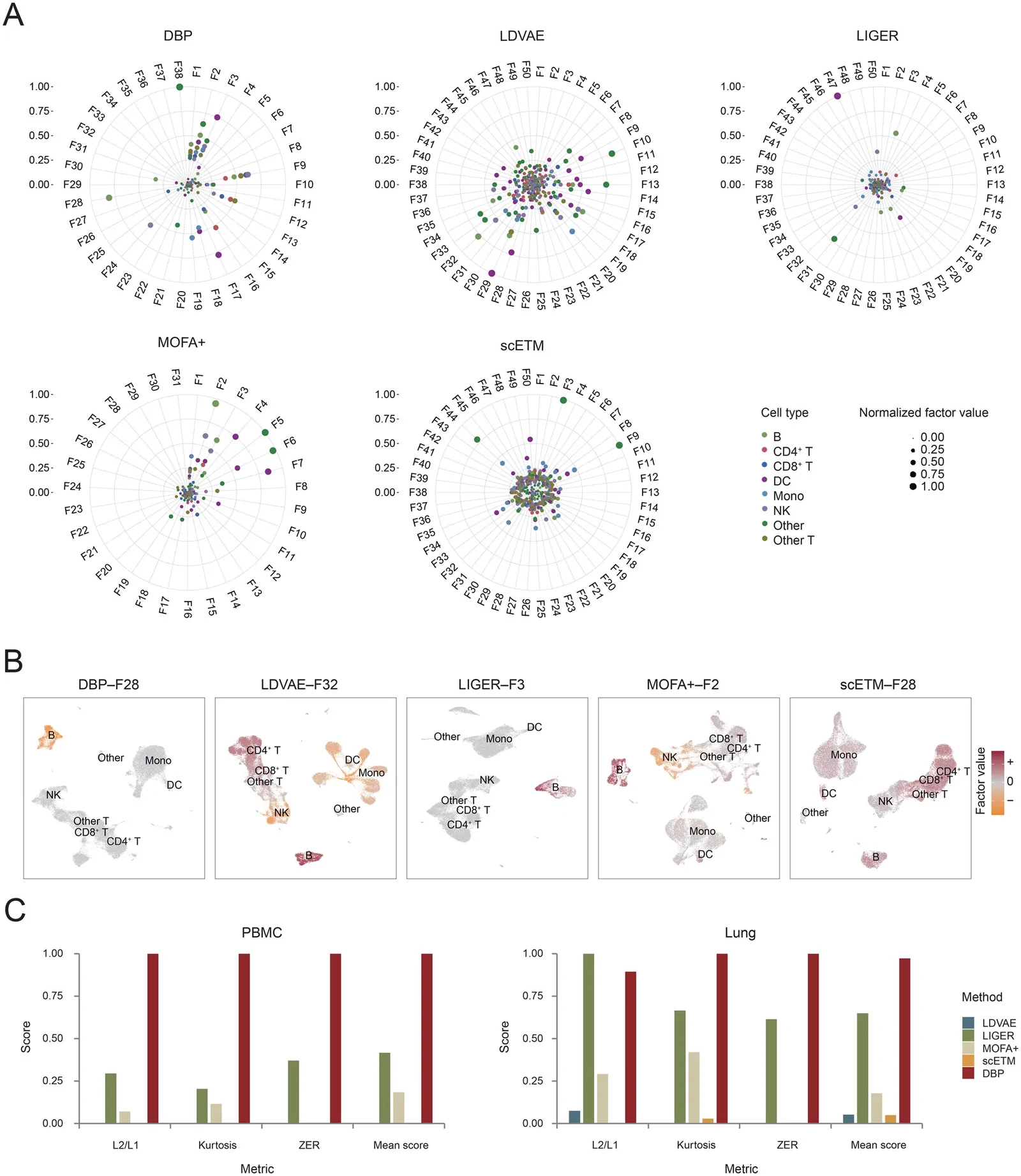
Evaluation of DBP performance on biological interpretability **A**. PBC visualization of the sparse factor matrix obtained by DBP and the cell embeddings obtained by four other methods in the PBMC dataset. The rays represent individual columns, while the circle radius and bubble size indicate the normalized mean column values for each cell type. **B**. UMAP visualization of the sparse factor values obtained by DBP and the cell embedding values obtained by other methods corresponding to B cells in the PBMC dataset. **C**. Benchmarking the interpretability performance of DBP and other methods on the PBMC and lung datasets. PBC, polar bubble chart; ZER, zero element ratio.

### DBP identifies cellular and genetic biosignatures

Understanding GPs in healthy individuals is crucial for investigating cellular and molecular heterogeneity. To explore the biological meaning of the sparse factors identified by DBP, we conducted downstream biological analyses on sparse factors extracted from both the PBMC and lung datasets. We classified these sparse factors into two categories: specific and shared. Specific sparse factors exhibit high factor values in individual cell types, capturing type-specific variations that define unique identities, such as CD4^+^ T cells. Shared sparse factors show elevated factor values across multiple cell types with similar expression patterns, reflecting biological processes like cell division and immune activation.

The results showed that DBP effectively extracted specific sparse factors. For instance, in the PBMC dataset, factors such as F17–DC, F19–Mono, F24–NK, F27–CD8^+^ T, and F28–B were effectively identified (**Figure 5**A). Additionally, DBP identified specific sparse factors representing cell types at a higher resolution, such as F6–Eryth, F21–cDC1, and F38–Platelet in the PBMC dataset, as well as subtypes in the lung dataset (Figure S5A and B). By examining the factor loadings associated with these specific sparse factors, we observed that genes with high factor loading values were related to cell identities (Figure 5B, Figure S5C). For instance, the dendritic cell (DC) marker *FCER1A* was highly expressed in the F17 factor in the PBMC dataset.

**Figure 5:**
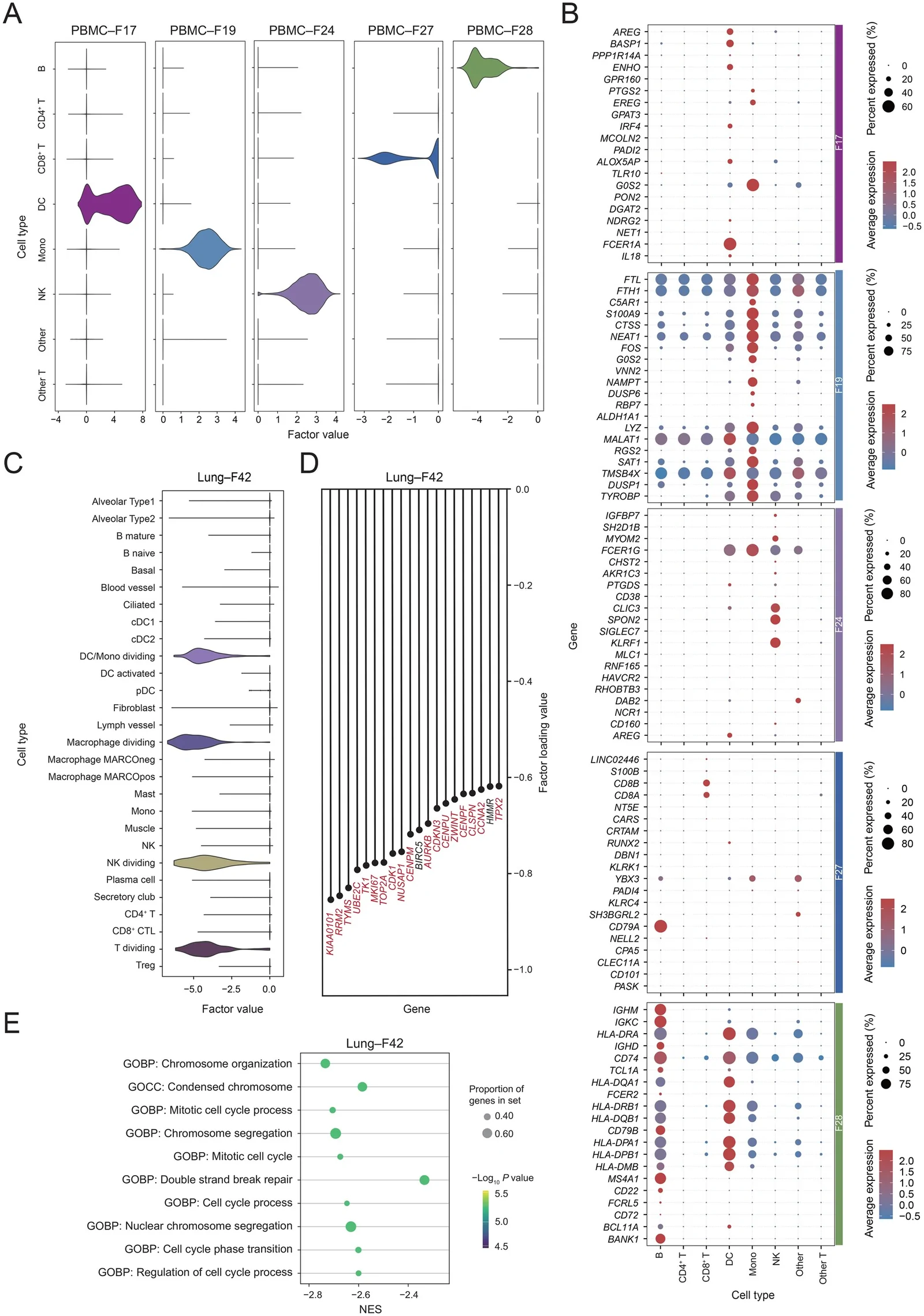
Interpretable sparse factors identified by DBP **A**. Violin plots showing the specific sparse factor values in the PBMC dataset. **B**. Dot plots showing the expression of the top 20 genes in five specific sparse factors (F17, F19, F24, F27, and F28) in the PBMC dataset. **C**. Violin plot showing the shared sparse factor values in the lung dataset. F42 shows higher sparse factor values in all dividing cells. **D**. Lollipop plot showing the factor loading values for the top 20 genes in Lung–F42. Genes related to cell cycle processes are shown in red. **E**. GSEA results of the factor loading vector for Lung–F42. The majority of genes are related to cell cycle processes. GOBP, Gene Ontology Biological Process; NES, Normalized Enrichment Score.

With the aid of GSEA, we identified the GPs associated with the shared sparse factors, which include F4–Chemotaxis, F7–Interferon response, and F10–Defense response to fungi or bacteria in the PBMC dataset (Tables S4 and S5), as well as F10–Extracellular matrix, F31–Cilium organization, and F32–DNA replication in the lung dataset (Tables S6 and S7). Remarkably, the GSEA results of both PBMC–F22 and Lung–F42 indicated their association with cell cycle processes (Figure 5C and E, Figure S5D). Moreover, the top 20 genes with high factor loading values were enriched in centromere protein (*CENP*) family genes, which are related to cell division (Figure 5D, Figure S5E). These results demonstrate the capability of DBP to extract interpretable GPs and establish correlations between cells and genes associated with these GPs.

Based on these fascinating findings, we delved deeper into the functional roles of genes with varying weights in the factor loadings. Our investigation uncovered that dysregulated expression of tail genes with lower factor loading values in normal cells played a crucial role in the initiation of specific diseases. For instance, we observed that the *IL*-*7R* gene in the PBMC–F24–NK tail genes showed upregulation in natural killer (NK) cells of multiple sclerosis patients [46]. Similarly, we found that aberrant expression of certain T cell markers (*CD3E* and *CD3G*) in the PBMC–F28–B tail genes was associated with diffuse large B cell lymphoma (Table S5) [47,48]. Through the analysis of the tail 20 genes of the Lung–F10–Extracellular matrix, we identified genes such as *RGS5*, *TM4SF1*, and *CXCL8*, which have been validated for their relevance to lung diseases (Table S7).

These findings suggest that the interpretable sparse factors extracted by DBP not only illuminate cellular heterogeneity and gene expression disparities but also offer valuable insights into the understanding of related disease mechanisms.

### Application of DBP to GA data

GA exhibits significant malignant epithelial cell heterogeneity, with diverse cell types possessing unique molecular characteristics within the same tumor. The varying sensitivity of these cells to treatment makes both accurate diagnosis and personalized therapy challenging. Given that DBP can decompose cellular heterogeneity into different sparse factors, we applied DBP to single-cell transcriptomic data of GA. The GA dataset used in this study was obtained from the same experimental batch with a reasonable experimental design. The UMAP visualization results suggested that the influence of batch differences between samples on biological variation was negligible (Figure S6A). Therefore, we excluded the batch correction module of DBP during the model training process.

In the GA dataset, DBP successfully identified sparse factors associated with the distinction of epithelial cells, classified as F18–Non-malignant epithelium, F23–Epithelium, and F28–Malignant epithelium, respectively (**Figure 6**A). By considering the gene weight information in the factor loading matrix, we also identified differentially expressed genes in non-malignant epithelium, such as *LIPF*, as well as genes differentially expressed in malignant epithelium, including *PI3*, *FABP1*, and *TM4SF20*, which are associated with tumor development (Figure 6B). Additionally, we discovered that F35 represented a B cell subtype with distinct expression of genes, such as *IGJ*, which has been shown to be instrumental in the differentiation of memory B cells to plasma cells [49] (Figure S6B and C; Table S8). These findings demonstrate the ability of DBP to explain malignant epithelial cell heterogeneity from both cellular and genetic perspectives.

**Figure 6:**
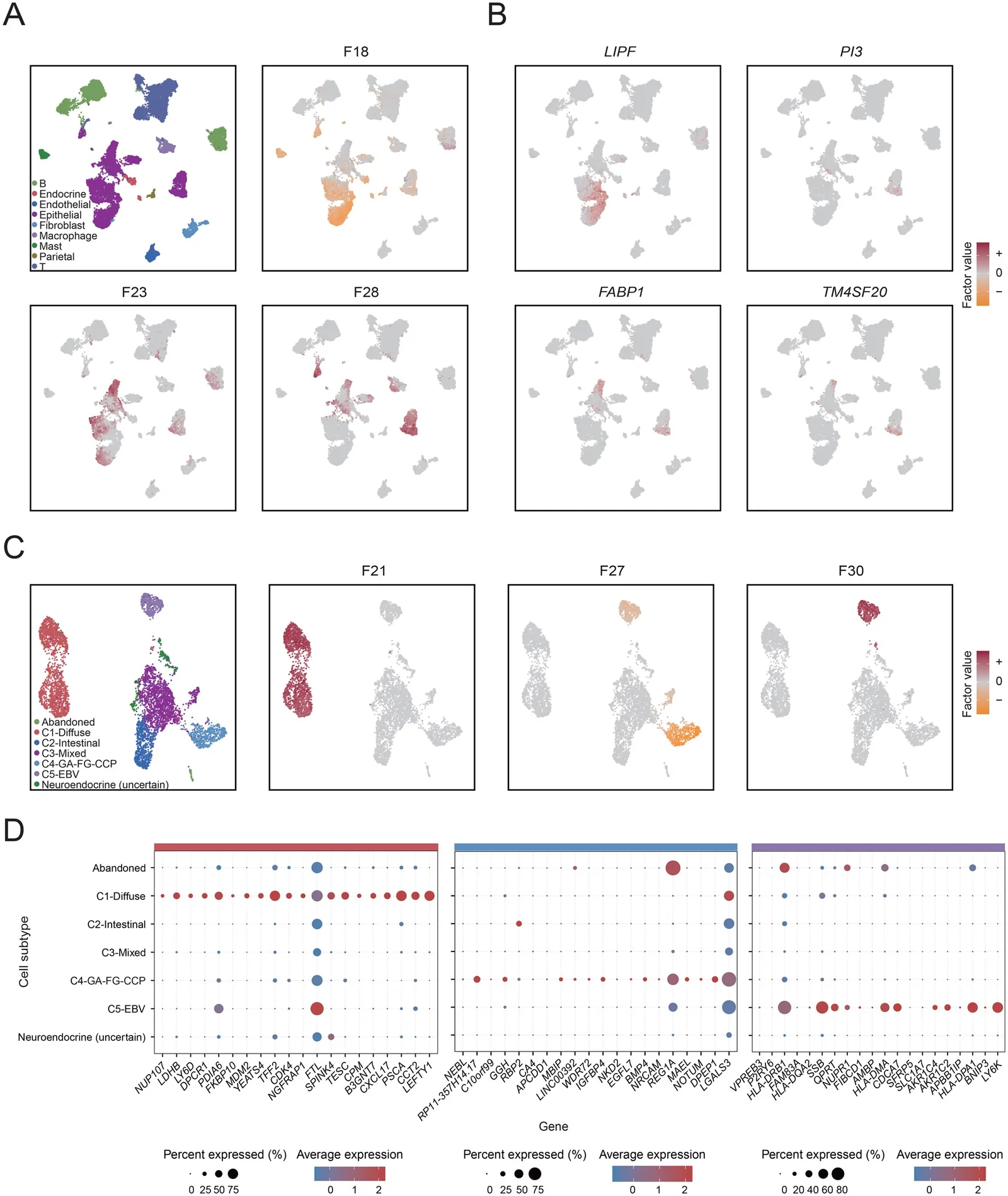
Application of DBP on the GA dataset **A**. UMAP visualization of the specific sparse factor values associated with epithelial cells learned by DBP in the GA dataset. The upper-left panel shows the sparse factor matrix obtained by DBP. **B**. Feature plots showing the differentially expressed genes in non-malignant cells (*LIPF*) and malignant epithelial cells (*PI3*, *FABP1*, and *TM4SF20*) identified by DBP. **C**. UMAP visualization of the specific sparse factor values representing cell subtypes identified by DBP in the GA_mali dataset. The left panel shows the sparse factor matrix obtained by DBP, while the three right panels show the sparse factor values of F21, F27, and F30, respectively. **D**. Dot plots showing the expression of the top 20 genes in three specific sparse factors (F21, F27, and F30) in the GA_mali dataset. GA, gastric adenocarcinoma; GA-FG-CCP, chief cell predominant GA; EBV, Epstein-Barr virus.

To investigate the heterogeneity of malignant tumor cells in greater detail, we isolated all malignant epithelial cells to obtain a dataset named GA_mali. Within the GA_mali dataset, we identified three factors, each representing a prominent cell subgroup. Specifically, F21 corresponds to cells in an undifferentiated state (C1), F27 corresponds to a rare variant of GA, namely GA-FG-CCP (C4), and F30 corresponds to cells from the Epstein-Barr virus (EBV)-positive patient (C5) (Figure 6C). Furthermore, we found that the top 20 genes in the factor loading vector of each factor were highly expressed in the corresponding cell subtypes (Figure 6D). Remarkably, F30–C5 exhibited elevated expression levels of immune response-related genes (*LY6K*, *HLA-DPA1*, *HLA-DQA2*, *HLA-DRB1*, and *HLA-DMA*), cell cycle regulation genes (*CDCA7*, *SSB*, and *NUPR1*), and genes encoding members of the aldo-keto reductase family (*AKR1C2* and *AKR1C4*). These findings demonstrate the upregulation of HLA class II genes, *LY6K*, and cell cycle regulatory genes in EBV-positive patients [50], suggesting a potential association between AKR1C superfamily proteins and NADPH metabolism in EBV-positive patients.

Thus, the application of DBP to tumor transcriptomic data facilitates the identification of novel cellular subtypes, elucidates the cellular and molecular characteristics of tumors and their subtypes, and provides comprehensive insights into the molecular mechanisms underlying tumor progression.

## Discussion

DBP combines factor analysis, stick-breaking Beta process, and adversarial learning to construct a deep generative model for single-cell transcriptomic data analysis. It can flexibly capture biological variation and effectively eliminate batch noise to obtain interpretable sparse factors. DBP shows superior performance in dimensionality reduction and factorization. By decomposing the gene expression matrix into sparse factor and factor loading matrices, DBP identifies data-driven sparse factors and infers the biological meanings of cells and genomic features. The application of DBP to highly heterogeneous GA transcriptomic datasets not only distinguishes clinically challenging tumor subtypes but also reveals the molecular characteristics of EBV-infected patients.

DBP uniquely determines the optimal number of sparse factors by considering the complexity and distribution of the observed data. Unlike other search-based methods, DBP avoids the need for manual selection and pruning, thereby eliminating intensive computations to obtain non-redundant GPs. Additionally, DBP effectively removes batch noise, which is crucial for ensuring interpretability.

We foresee several potential extensions of adaptive factor analysis methods in the future. First, the abundance of intricate and complex cellular state information in single-cell data poses significant challenges for the comprehensive and accurate annotation of GPs. To address this challenge, incorporating a larger cohort of cells from diverse species could provide a broader and more nuanced understanding of interpretable sparse factors. Second, with the continuous accumulation of new single-cell transcriptomic data, there is an increasing demand for adaptive factor analysis methods capable of handling large-scale atlas-level data. Such methods should be designed to integrate a wide range of biological features across different scales, facilitating the systematic exploration of cellular mechanisms.

## Code availability

DBP was implemented using the Python (v3.8.8) package PyTorch (v2.0.0) with code available at https://github.com/labomics/DBP. DBP has also been submitted to BioCode at the National Genomics Data Center (NGDC), China National Center for Bioinformation (CNCB) (BioCode: BT007954), which is publicly accessible at https://ngdc.cncb.ac.cn/biocode/tool/BT007954.

## CRediT author statement

**Runyan Liu:** Conceptualization, Data curation, Formal analysis, Methodology, Software, Visualization, Writing – original draft, Writing – review & editing. **Shuofeng Hu: **Data curation, Formal analysis. **Guohua Dong:** Visualization. **Tongtong Kan:** Data curation. **Jinhui Shi:** Formal analysis. **Jing Wang:** Data curation. **Jiahao Zhou:** Data curation. **Zhen He:** Conceptualization, Methodology, Resources, Supervision, Writing – original draft, Writing – review & editing. **Xiaomin Ying:** Conceptualization, Investigation, Project administration, Resources, Supervision, Writing – original draft, Writing – review & editing. All authors have read and approved the final manuscript.

## Competing interests

The authors have declared no competing interests.

## Supplementary Material

qzaf117_Supplementary_Data
