# Supplementary material for: DBP: Adaptive and Interpretable Factor Analysis for Single-cell RNA-seq Data with Deep Beta Processes
Source: Genomics Proteomics Bioinformatics. 2025 Nov 26;24(3):qzaf117. doi: 10.1093/gpbjnl/qzaf117 (PMC13686767; doi:10.1093/gpbjnl/qzaf117)
Supplement: Supplementary file 1 [file qzaf117_supplementary_data.zip › qzaf117_Supplementary_data-new.zip!/Figure S3.pdf]

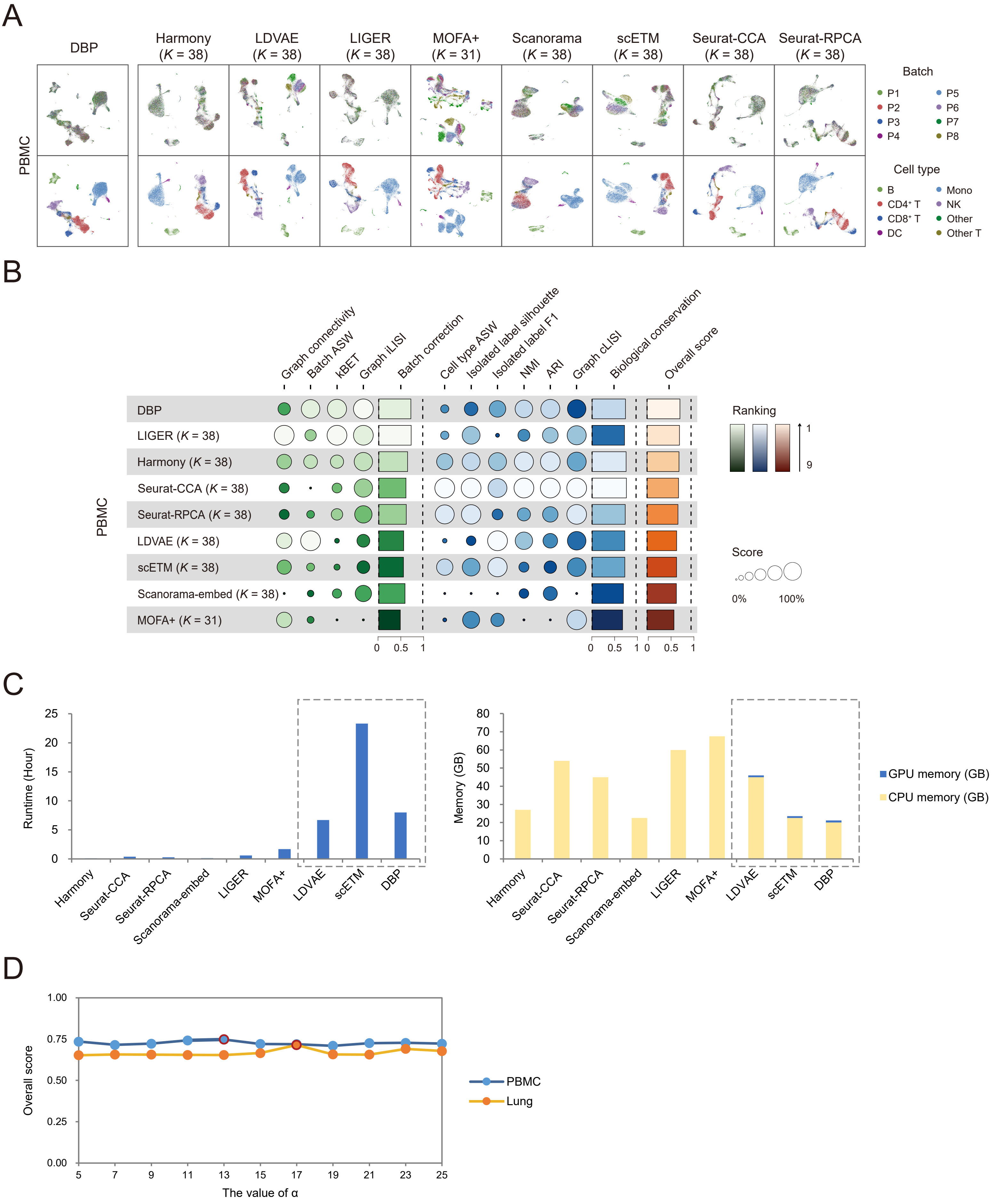
A
DBP
Harmony (K = 38)
LDVAE (K = 38)
LIGER (K = 38)
MOFA+ (K = 31)
Scanorama (K = 38)
scETM (K = 38)
Seurat-CCA (K = 38)
Seurat-RPCA (K = 38)
PBMC
Batch
P1
P2
P3
P4
P5
P6
P7
P8
Cell type
B
CD4⁺ T
CD8⁺ T
DC
Mono
NK
Other
Other T
B
Graph connectivity
Batch ASW
kBET
Graph iLISI
Batch correction
Cell type ASW
Isolated label silhouette
Isolated label F1
NMI
ARI
Graph cLISI
Biological conservation
Overall score
DBP
LIGER (K = 38)
Harmony (K = 38)
Seurat-CCA (K = 38)
Seurat-RPCA (K = 38)
LDVAE (K = 38)
scETM (K = 38)
Scanorama-embed (K = 38)
MOFA+ (K = 31)
PBMC
Ranking
1
9
Score
0%
100%
0 0.5 1
0 0.5 1
0 0.5 1
C
Runtime (Hour)
25
20
15
10
5
0
Harmony
Seurat-CCA
Seurat-RPCA
Scanorama-embed
LIGER
MOFA+
LDVAE
scETM
DBP
Memory (GB)
80
70
60
50
40
30
20
10
0
GPU memory (GB)
CPU memory (GB)
D
Overall score
1.00
0.75
0.50
0.25
0.00
5 7 9 11 13 15 17 19 21 23 25
The value of α
PBMC
Lung
