# Supplementary material for: DBP: Adaptive and Interpretable Factor Analysis for Single-cell RNA-seq Data with Deep Beta Processes
Source: Genomics Proteomics Bioinformatics. 2025 Nov 26;24(3):qzaf117. doi: 10.1093/gpbjnl/qzaf117 (PMC13686767; doi:10.1093/gpbjnl/qzaf117)
Supplement: Supplementary file 1 [file qzaf117_supplementary_data.zip › qzaf117_Supplementary_data-new.zip!/Figure S4.pdf]

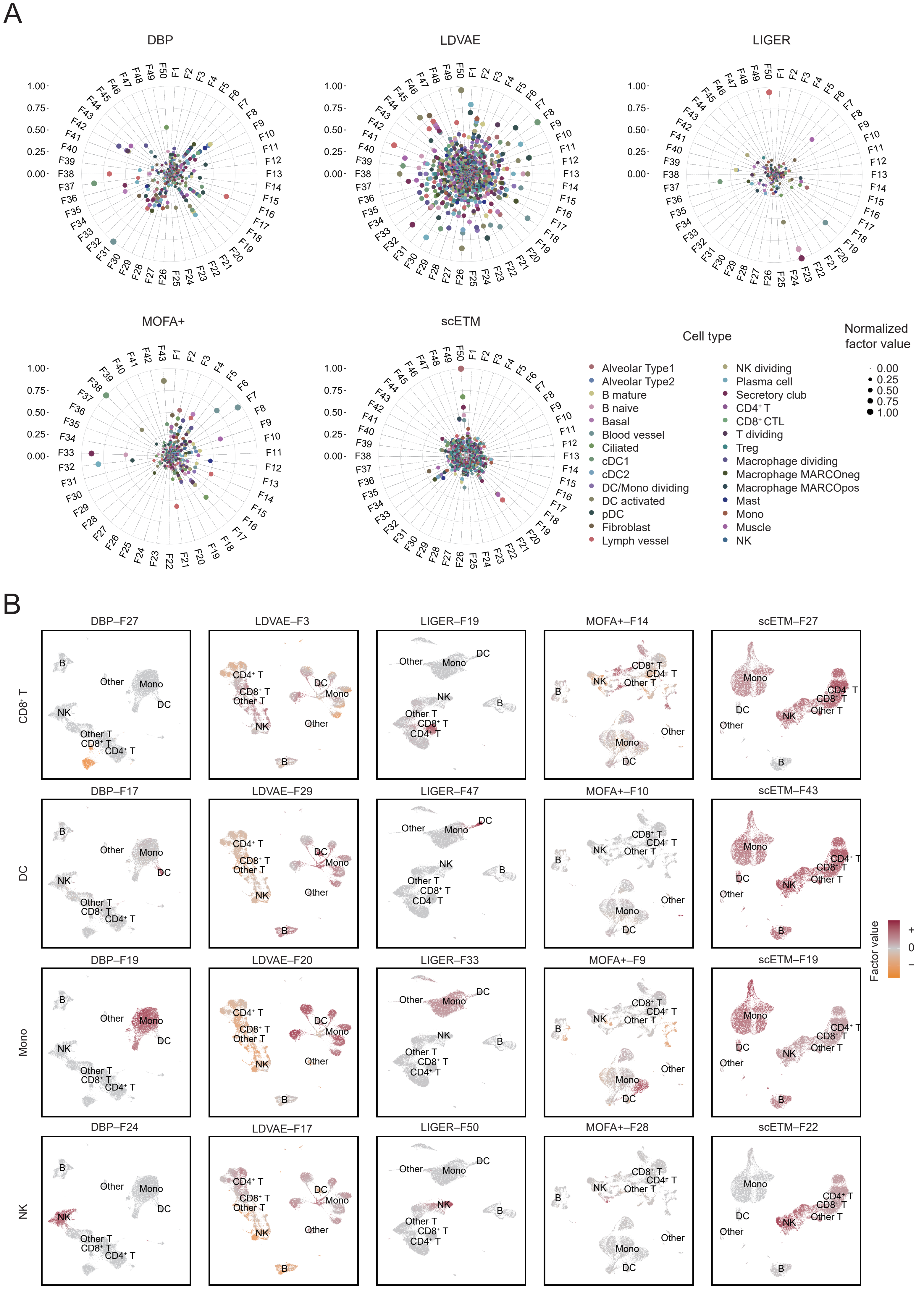
A
DBP
LDVAE
LIGER
MOFA+
scETM
1.00
0.75
0.50
0.25
0.00
Cell type
Alveolar Type1
Alveolar Type2
B mature
B naive
Basal
Blood vessel
Ciliated
cDC1
cDC2
DC/Mono dividing
DC activated
pDC
Fibroblast
Lymph vessel
NK dividing
Plasma cell
Secretory club
CD4⁺ T
CD8⁺ CTL
T dividing
Treg
Macrophage dividing
Macrophage MARCOneg
Macrophage MARCOpos
Mast
Mono
Muscle
NK
Normalized factor value
0.00
0.25
0.50
0.75
1.00
B
DBP–F27
LDVAE–F3
LIGER–F19
MOFA+–F14
scETM–F27
DBP–F17
LDVAE–F29
LIGER–F47
MOFA+–F10
scETM–F43
DBP–F19
LDVAE–F20
LIGER–F33
MOFA+–F9
scETM–F19
DBP–F24
LDVAE–F17
LIGER–F50
MOFA+–F28
scETM–F22
CD8⁺ T
DC
Mono
NK
Factor value
+
0
–
