# Supplementary material for: DBP: Adaptive and Interpretable Factor Analysis for Single-cell RNA-seq Data with Deep Beta Processes
Source: Genomics Proteomics Bioinformatics. 2025 Nov 26;24(3):qzaf117. doi: 10.1093/gpbjnl/qzaf117 (PMC13686767; doi:10.1093/gpbjnl/qzaf117)
Supplement: Supplementary file 1 [file qzaf117_supplementary_data.zip › qzaf117_Supplementary_data-new.zip!/Figure S5.pdf]

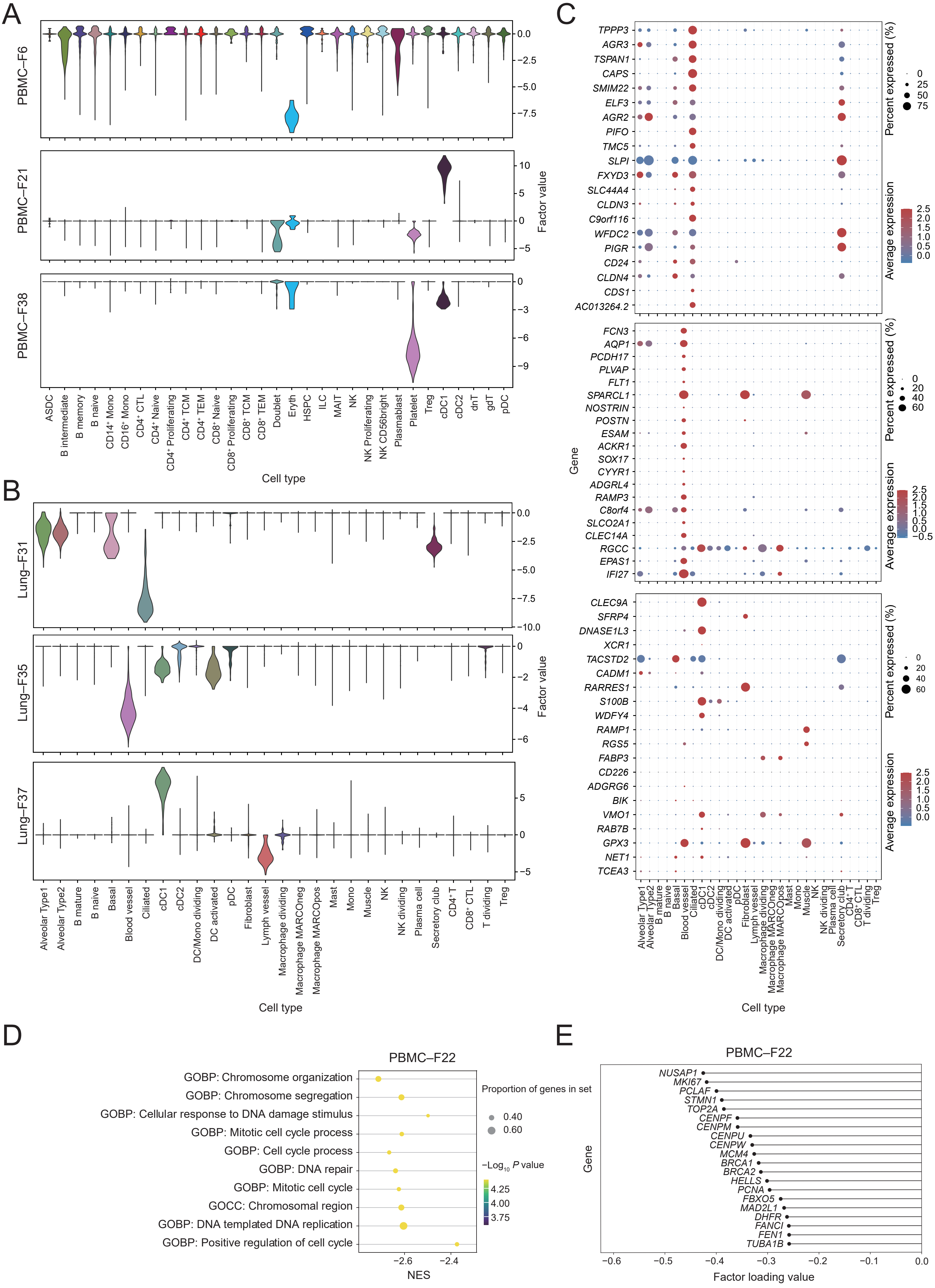

A
PBMC–F6
PBMC–F21
PBMC–F38
Factor value
ASDC
B intermediate
B memory
B naive
CD14+ Mono
CD16+ Mono
CD4+ CTL
CD4+ Naive
CD4+ Proliferating
CD4+ TCM
CD4+ TEM
CD8+ Naive
CD8+ Proliferating
CD8+ TCM
CD8+ TEM
Doublet
Eryth
HSPC
ILC
MAIT
NK
NK Proliferating
NK CD56bright
Plasmablast
Platelet
Treg
cDC1
cDC2
dnT
gdT
pDC
Cell type
B
Lung–F31
Lung–F35
Lung–F37
Factor value
Alveolar Type1
Alveolar Type2
B mature
B naive
Basal
Blood vessel
Ciliated
cDC1
cDC2
DC/Mono dividing
DC activated
pDC
Fibroblast
Lymph vessel
Macrophage dividing
Macrophage MARCOneg
Macrophage MARCOpos
Mast
Mono
Muscle
NK
NK dividing
Plasma cell
Secretory club
CD4+ T
CD8+ CTL
T dividing
Treg
Cell type
C
TPPP3
AGR3
TSPAN1
CAPS
SMIM22
ELF3
AGR2
PIFO
TMC5
SLPI
FXYD3
SLC44A4
CLDN3
C9orf116
WFDC2
PIGR
CD24
CLDN4
CDS1
AC013264.2
FCN3
AQP1
PCDH17
PLVAP
FLT1
SPARCL1
NOSTRIN
POSTN
ESAM
ACKR1
SOX17
CYYR1
ADGRL4
RAMP3
C8orf4
SLCO2A1
CLEC14A
RGCC
EPAS1
IFI27
CLEC9A
SFRP4
DNASE1L3
XCR1
TACSTD2
CADM1
RARRES1
S100B
WDFY4
RAMP1
RGS5
FABP3
CD226
ADGRG6
BIK
VMO1
RAB7B
GPX3
NET1
TCEA3
Gene
Percent expressed (%)
Average expression
Cell type
D
PBMC–F22
GOBP: Chromosome organization
GOBP: Chromosome segregation
GOBP: Cellular response to DNA damage stimulus
GOBP: Mitotic cell cycle process
GOBP: Cell cycle process
GOBP: DNA repair
GOBP: Mitotic cell cycle
GOCC: Chromosomal region
GOBP: DNA templated DNA replication
GOBP: Positive regulation of cell cycle
NES
Proportion of genes in set
0.40
0.60
–Log10 P value
E
PBMC–F22
NUSAP1
MKI67
PCLAF
STMN1
TOP2A
CENPF
CENPM
CENPU
CENPW
MCM4
BRCA1
BRCA2
HELLS
PCNA
FBXO5
MAD2L1
DHFR
FANCI
FEN1
TUBA1B
Gene
Factor loading value
