# Supplementary material for: DBP: Adaptive and Interpretable Factor Analysis for Single-cell RNA-seq Data with Deep Beta Processes
Source: Genomics Proteomics Bioinformatics. 2025 Nov 26;24(3):qzaf117. doi: 10.1093/gpbjnl/qzaf117 (PMC13686767; doi:10.1093/gpbjnl/qzaf117)
Supplement: Supplementary file 1 [file qzaf117_supplementary_data.zip › qzaf117_Supplementary_data-new.zip!/Figure S6.pdf]

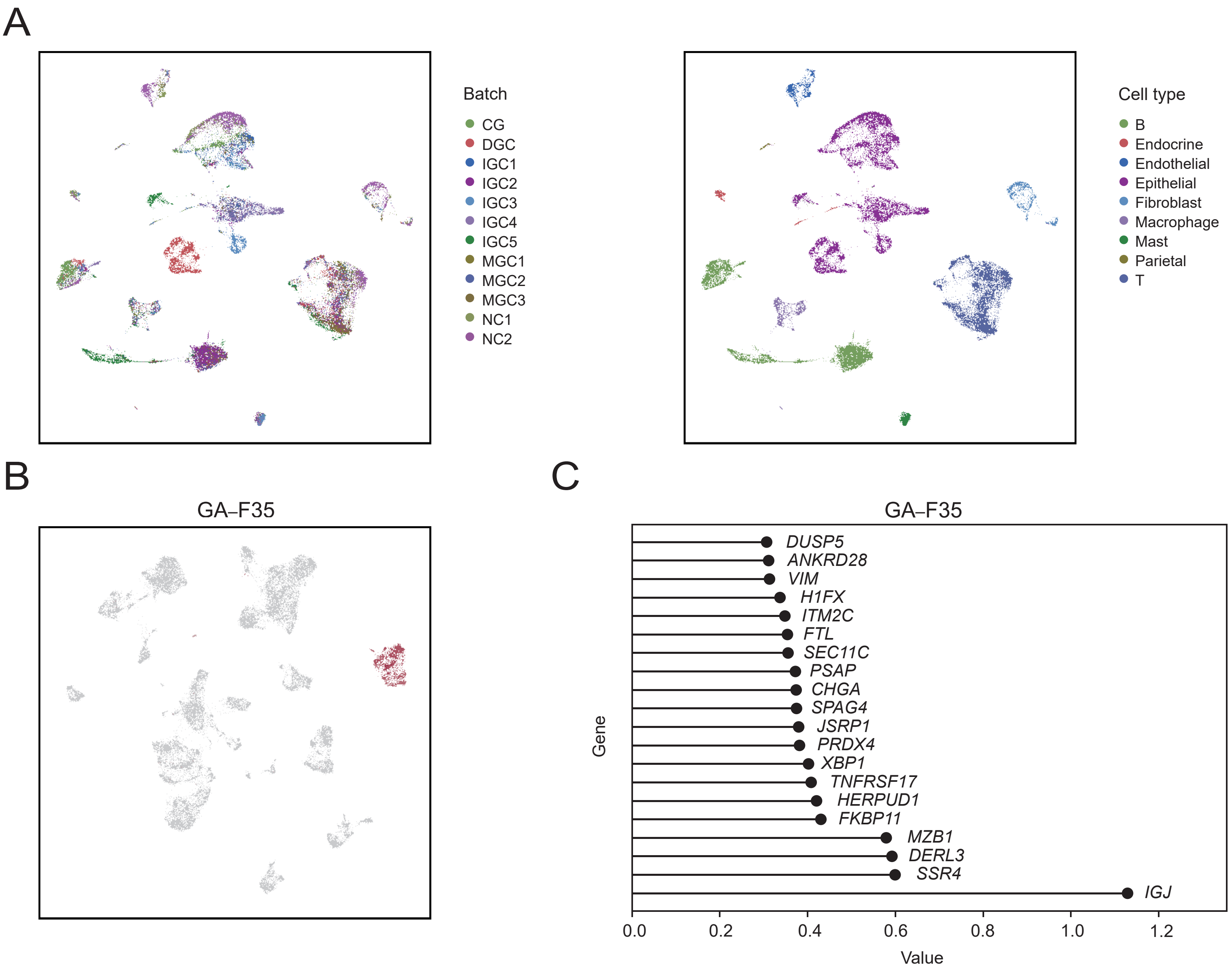
A
Batch
CG
DGC
IGC1
IGC2
IGC3
IGC4
IGC5
MGC1
MGC2
MGC3
NC1
NC2
Cell type
B
Endocrine
Endothelial
Epithelial
Fibroblast
Macrophage
Mast
Parietal
T
B
GA–F35
C
GA–F35
Gene
DUSP5
ANKRD28
VIM
H1FX
ITM2C
FTL
SEC11C
PSAP
CHGA
SPAG4
JSRP1
PRDX4
XBP1
TNFRSF17
HERPUD1
FKBP11
MZB1
DERL3
SSR4
IGJ
0.0
0.2
0.4
0.6
0.8
1.0
1.2
Value
