# Supplementary material for: DBP: Adaptive and Interpretable Factor Analysis for Single-cell RNA-seq Data with Deep Beta Processes
Source: Genomics Proteomics Bioinformatics. 2025 Nov 26;24(3):qzaf117. doi: 10.1093/gpbjnl/qzaf117 (PMC13686767; doi:10.1093/gpbjnl/qzaf117)
Supplement: Supplementary file 1 [file qzaf117_supplementary_data.zip › qzaf117_Supplementary_data-new.zip!/File S1.docx]

**File S1 Derivation of the generative process**

The detailed derivation of the generative process for the observed variables $\boldsymbol{X}$ and $\boldsymbol{s}$ of Equation 1 is as follows:

$$\begin{aligned} p\left( \boldsymbol{X}, \boldsymbol{s}, \hat{\boldsymbol{\beta}}, \boldsymbol{W}, \boldsymbol{C}, \boldsymbol{B}, \boldsymbol{A} \right)&=p\left( \hat{\boldsymbol{\beta}}, \boldsymbol{A} \right)p\left( \boldsymbol{X}, \boldsymbol{s}, \boldsymbol{W},\boldsymbol{C}, \boldsymbol{B} | \hat{\boldsymbol{\beta}}, \boldsymbol{A} \right)\#\# \\ &=p\left( \hat{\boldsymbol{\beta}} \right)p\left( \boldsymbol{A} \right)p\left( \boldsymbol{X}, \boldsymbol{s}, \boldsymbol{W},\boldsymbol{C}, \boldsymbol{B} | \hat{\boldsymbol{\beta}}, \boldsymbol{A} \right) \\ &=p\left( \hat{\boldsymbol{\beta}} \right)p\left( \boldsymbol{A} \right)p\left( \boldsymbol{W} | \hat{\boldsymbol{\beta}} \right)p\left( \boldsymbol{C} \right)p\left( \boldsymbol{B} \right)p\left( \boldsymbol{s} | \boldsymbol{B} \right)p\left( \boldsymbol{X} | \boldsymbol{W}, \boldsymbol{C}, \boldsymbol{B}, \boldsymbol{A} \right)\#\#(14) \\ &=\prod_{k=1}^{\infty} p\left( \hat{\beta}_{k} \right)p\left( \boldsymbol{a}_{k} \right)\prod_{n=1}^{N} p\left( \boldsymbol{w}_{n} | \hat{\boldsymbol{\beta}} \right)p\left( \boldsymbol{c}_{n} \right)p\left( \boldsymbol{b}_{n} \right)p\left( s_{n} | \boldsymbol{b}_{n} \right)p\left( \boldsymbol{x}_{n} | \boldsymbol{w}_{n}, \boldsymbol{c}_{n}, \boldsymbol{b}_{n},\boldsymbol{A} \right)\#\# \end{aligned}$$

By leveraging the chain rule of probability and conditional independence assumptions, the distribution is factorized into a product of simpler terms. Specifically, $\hat{\boldsymbol{\beta}}$ and $\boldsymbol{A}$ are global latent variables with their dimensions $\hat{\beta}_{k}$ and $\boldsymbol{a}_{k}$ being mutually independent across factors $k$.
