# Supplementary material for: DBP: Adaptive and Interpretable Factor Analysis for Single-cell RNA-seq Data with Deep Beta Processes
Source: Genomics Proteomics Bioinformatics. 2025 Nov 26;24(3):qzaf117. doi: 10.1093/gpbjnl/qzaf117 (PMC13686767; doi:10.1093/gpbjnl/qzaf117)
Supplement: Supplementary file 1 [file qzaf117_supplementary_data.zip › qzaf117_Supplementary_data-new.zip!/File S2.docx]

**File S2 Derivation of the ELBO**

The detailed derivation of the ELBO for Equation 4 is as follows:

$$\text{ELBO}(\boldsymbol{\theta},\boldsymbol{\phi};\boldsymbol{X},\boldsymbol{s})$$

$$\triangleq\mathbb{E}_{q_{\boldsymbol{\phi}}\mathcal{(Z|}\boldsymbol{X},\boldsymbol{s})}\left[ {\log p}_{\boldsymbol{\theta}}(\boldsymbol{X},\boldsymbol{s}\mathcal{|Z)} \right]-\text{KL}\left( q_{\boldsymbol{\phi}}\mathcal{(Z|}\boldsymbol{X},\boldsymbol{s})\|p(\mathcal{Z)} \right)$$

$$=\mathbb{E}_{q_{\boldsymbol{\phi}}\mathcal{(Z|}\boldsymbol{X},\boldsymbol{s})}[{\log p}_{\boldsymbol{\theta}}(\boldsymbol{X},\boldsymbol{s}\mathcal{|Z)]}-\mathbb{E}_{q_{\boldsymbol{\phi}}\mathcal{(Z|}\boldsymbol{X},\boldsymbol{s})}\left[ {\log q}_{\boldsymbol{\phi}}\mathcal{(Z|}\boldsymbol{X},\boldsymbol{s})-\log p\mathcal{(Z)} \right]$$

$$=\mathbb{E}_{q_{\boldsymbol{\phi}}\mathcal{(Z|}\boldsymbol{X},\boldsymbol{s})}\left[ {\log p}_{\boldsymbol{\theta}}(\boldsymbol{X},\boldsymbol{s}\mathcal{,Z)} \right]-\mathbb{E}_{q_{\boldsymbol{\phi}}\mathcal{(Z|}\boldsymbol{X},\boldsymbol{s})}\left[ {\log q}_{\boldsymbol{\phi}}\mathcal{(Z|}\boldsymbol{X},\boldsymbol{s}) \right]$$

$$=\mathbb{E}_{q_{\boldsymbol{\phi}}(\hat{\boldsymbol{\beta}},\boldsymbol{W},\boldsymbol{C},\boldsymbol{B},\boldsymbol{A}|\boldsymbol{X},\boldsymbol{s})}\left[ \underset{={\log p}_{\boldsymbol{\theta}}(\boldsymbol{s}|\boldsymbol{B})+\log p(\boldsymbol{X},\hat{\boldsymbol{\beta}},\boldsymbol{W},\boldsymbol{C},\boldsymbol{B},\boldsymbol{A})}{\underbrace{{\log p}_{\boldsymbol{\theta}}(\boldsymbol{X},\boldsymbol{s},\hat{\boldsymbol{\beta}},\boldsymbol{W},\boldsymbol{C},\boldsymbol{B},\boldsymbol{A})}} \right]-\mathbb{E}_{q_{\boldsymbol{\phi}}(\hat{\boldsymbol{\beta}},\boldsymbol{W},\boldsymbol{C},\boldsymbol{B},\boldsymbol{A}|\boldsymbol{X},\boldsymbol{s})}\left[ {\log q}_{\boldsymbol{\phi}}(\hat{\boldsymbol{\beta}},\boldsymbol{W},\boldsymbol{C},\boldsymbol{B},\boldsymbol{A}|\boldsymbol{X},\boldsymbol{s}) \right]$$

$$=\mathbb{E}_{q_{\boldsymbol{\phi}}(\hat{\boldsymbol{\beta}},\boldsymbol{W},\boldsymbol{C},\boldsymbol{B},\boldsymbol{A}|\boldsymbol{X},\boldsymbol{s})}\left[ \begin{aligned} \#\log p\left( \boldsymbol{X} | \boldsymbol{W},\boldsymbol{C},\boldsymbol{B},\boldsymbol{A} \right)+{\log p}_{\boldsymbol{\theta}}\left( \boldsymbol{s} | \boldsymbol{B} \right)+\log p\left( \hat{\boldsymbol{\beta}} \right)+\log p\left( \boldsymbol{W} | \hat{\boldsymbol{\beta}} \right)+\log p\left( \boldsymbol{C} \right) \\ \#+\log p(\boldsymbol{B})+\log p(\boldsymbol{A}) \end{aligned} \right]$$

$$-\mathbb{E}_{q_{\boldsymbol{\phi}}\left( \hat{\boldsymbol{\beta}},\boldsymbol{W},\boldsymbol{C},\boldsymbol{B},\boldsymbol{A} | \boldsymbol{X},\boldsymbol{s} \right)}\left[ \begin{aligned} \#{\log q}_{\boldsymbol{\phi}}\left( \hat{\boldsymbol{\beta}} | \boldsymbol{X},\boldsymbol{s} \right)+{\log q}_{\boldsymbol{\phi}}\left( \boldsymbol{W} | \boldsymbol{X},\boldsymbol{s},\hat{\boldsymbol{\beta}} \right)+{\log q}_{\boldsymbol{\phi}}\left( \boldsymbol{C} | \boldsymbol{X},\boldsymbol{s} \right) \\ \#\#+{\log q}_{\boldsymbol{\phi}}\left( \boldsymbol{B} | \boldsymbol{X},\boldsymbol{s} \right)+{\log q}_{\boldsymbol{\phi}}(\boldsymbol{A}|\boldsymbol{X},\boldsymbol{s}) \end{aligned} \right]$$

$$=\mathbb{E}_{q_{\boldsymbol{\phi}}(\hat{\boldsymbol{\beta}}|\boldsymbol{X},\boldsymbol{s})}\mathbb{E}_{q_{\boldsymbol{\phi}}(\boldsymbol{W}|\boldsymbol{X},\boldsymbol{s},\hat{\boldsymbol{\beta}})}\mathbb{E}_{q_{\boldsymbol{\phi}}(\boldsymbol{C}|\boldsymbol{X},\boldsymbol{s})}\mathbb{E}_{q_{\boldsymbol{\phi}}(\boldsymbol{B}|\boldsymbol{X},\boldsymbol{s})}\mathbb{E}_{q_{\boldsymbol{\phi}}(\boldsymbol{A}|\boldsymbol{X},\boldsymbol{s})}\left[ \log p(\boldsymbol{X}|\boldsymbol{W},\boldsymbol{C},\boldsymbol{B},\boldsymbol{A}) \right]$$

$$+\mathbb{E}_{q_{\boldsymbol{\phi}}(\boldsymbol{B}|\boldsymbol{X},\boldsymbol{s})}\left[ {\log p}_{\boldsymbol{\theta}}(\boldsymbol{s}|\boldsymbol{B}) \right]+\mathbb{E}_{q_{\boldsymbol{\phi}}(\hat{\boldsymbol{\beta}}|\boldsymbol{X},\boldsymbol{s})}\left[ \log p(\hat{\boldsymbol{\beta}}) \right]+\mathbb{E}_{q_{\boldsymbol{\phi}}(\hat{\boldsymbol{\beta}}|\boldsymbol{X},\boldsymbol{s})}\mathbb{E}_{q_{\boldsymbol{\phi}}(\boldsymbol{W}|\boldsymbol{X},\boldsymbol{s},\hat{\boldsymbol{\beta}})}\left[ \log p(\boldsymbol{W}|\hat{\boldsymbol{\beta}}) \right]$$

$$+\mathbb{E}_{q_{\boldsymbol{\phi}}(\boldsymbol{C}|\boldsymbol{X},\boldsymbol{s})}\left[ \log p(\boldsymbol{C}) \right]+\mathbb{E}_{q_{\boldsymbol{\phi}}(\boldsymbol{B}|\boldsymbol{X},\boldsymbol{s})}\left[ \log p(\boldsymbol{B}) \right]+\mathbb{E}_{q_{\boldsymbol{\phi}}(\boldsymbol{A}|\boldsymbol{X},\boldsymbol{s})}\left[ \log p(\boldsymbol{A}) \right]$$

$$-\mathbb{E}_{q_{\boldsymbol{\phi}}(\hat{\boldsymbol{\beta}}|\boldsymbol{X},\boldsymbol{s})}\left[ {\log q}_{\boldsymbol{\phi}}(\hat{\boldsymbol{\beta}}|\boldsymbol{X},\boldsymbol{s}) \right]-\mathbb{E}_{q_{\boldsymbol{\phi}}(\hat{\boldsymbol{\beta}}|\boldsymbol{X},\boldsymbol{s})}\mathbb{E}_{q_{\boldsymbol{\phi}}(\boldsymbol{W}|\boldsymbol{X},\boldsymbol{s},\hat{\boldsymbol{\beta}})}\left[ {\log q}_{\boldsymbol{\phi}}(\boldsymbol{W}|\boldsymbol{X},\boldsymbol{s},\hat{\boldsymbol{\beta}}) \right]$$

$$-\mathbb{E}_{q_{\boldsymbol{\phi}}(\boldsymbol{C}|\boldsymbol{X},\boldsymbol{s})}\left[ {\log q}_{\boldsymbol{\phi}}(\boldsymbol{C}|\boldsymbol{X},\boldsymbol{s}) \right]-\mathbb{E}_{q_{\boldsymbol{\phi}}(\boldsymbol{B}|\boldsymbol{X},\boldsymbol{s})}\left[ {\log q}_{\boldsymbol{\phi}}(\boldsymbol{B}|\boldsymbol{X},\boldsymbol{s}) \right]-\mathbb{E}_{q_{\boldsymbol{\phi}}(\boldsymbol{A}|\boldsymbol{X},\boldsymbol{s})}\left[ {\log q}_{\boldsymbol{\phi}}(\boldsymbol{A}|\boldsymbol{X},\boldsymbol{s}) \right]$$

$$=\mathbb{E}_{q_{\boldsymbol{\phi}}(\hat{\boldsymbol{\beta}}|\boldsymbol{X},\boldsymbol{s})}\mathbb{E}_{q_{\boldsymbol{\phi}}(\boldsymbol{W}|\boldsymbol{X},\boldsymbol{s},\hat{\boldsymbol{\beta}})}\mathbb{E}_{q_{\boldsymbol{\phi}}(\boldsymbol{C}|\boldsymbol{X},\boldsymbol{s})}\mathbb{E}_{q_{\boldsymbol{\phi}}(\boldsymbol{B}|\boldsymbol{X},\boldsymbol{s})}\mathbb{E}_{q_{\boldsymbol{\phi}}(\boldsymbol{A}|\boldsymbol{X},\boldsymbol{s})}\left[ \log p(\boldsymbol{X}|\boldsymbol{W},\boldsymbol{C},\boldsymbol{B},\boldsymbol{A}) \right]$$

$$+\mathbb{E}_{q_{\boldsymbol{\phi}}(\boldsymbol{B}|\boldsymbol{X},\boldsymbol{s})}\left[ {\log p}_{\boldsymbol{\theta}}(\boldsymbol{s}|\boldsymbol{B}) \right]-\mathrm{KL} \left( q_{\boldsymbol{\phi}}(\hat{\boldsymbol{\beta}}|\boldsymbol{X},\boldsymbol{s})\|p(\hat{\boldsymbol{\beta}}) \right)-\mathbb{E}_{q_{\boldsymbol{\phi}}(\hat{\boldsymbol{\beta}}|\boldsymbol{X},\boldsymbol{s})}\left[ \mathrm{KL} \left( q_{\boldsymbol{\phi}}(\boldsymbol{W}|\boldsymbol{X},\boldsymbol{s},\hat{\boldsymbol{\beta}})\|p(\boldsymbol{W}|\hat{\boldsymbol{\beta}}) \right) \right]$$

$$-\mathrm{KL} \left( q_{\boldsymbol{\phi}}(\boldsymbol{C}|\boldsymbol{X},\boldsymbol{s})\|p(\boldsymbol{C}) \right)-\text{KL}\left( q_{\boldsymbol{\phi}}(\boldsymbol{B}|\boldsymbol{X},\boldsymbol{s})\|p(\boldsymbol{B}) \right)-\text{KL}\left( q_{\boldsymbol{\phi}}(\boldsymbol{A}|\boldsymbol{X},\boldsymbol{s})\|p(\boldsymbol{A}) \right)$$

$$=\sum_{n=1}^{N} \left[ \begin{aligned} \#\mathbb{E}_{q_{\boldsymbol{\phi}}\left( \hat{\boldsymbol{\beta}} | \boldsymbol{X},\boldsymbol{s} \right)}\mathbb{E}_{q_{\boldsymbol{\phi}}\left( \boldsymbol{w}_{n} | \boldsymbol{x}_{n},s_{n},\hat{\boldsymbol{\beta}} \right)}\mathbb{E}_{q_{\boldsymbol{\phi}}\left( \boldsymbol{c}_{n} | \boldsymbol{x}_{n},s_{n} \right)}\mathbb{E}_{q_{\boldsymbol{\phi}}\left( \boldsymbol{b}_{n} | \boldsymbol{x}_{n},s_{n} \right)}\mathbb{E}_{q_{\boldsymbol{\phi}}\left( \boldsymbol{A} | \boldsymbol{X},\boldsymbol{s} \right)}\left[ \log p\left( \boldsymbol{x}_{n} | \boldsymbol{w}_{n},\boldsymbol{c}_{n},\boldsymbol{b}_{n},\boldsymbol{A} \right) \right] \\ \#+\mathbb{E}_{q_{\boldsymbol{\phi}}\left( \boldsymbol{b}_{n} | \boldsymbol{x}_{n},s_{n} \right)}\left[ {\log p}_{\boldsymbol{\theta}}\left( s_{n} | \boldsymbol{b}_{n} \right) \right]-\mathbb{E}_{q_{\boldsymbol{\phi}}\left( \hat{\boldsymbol{\beta}} | \boldsymbol{X},\boldsymbol{s} \right)}\left[ \mathrm{KL} \left( q_{\boldsymbol{\phi}}(\boldsymbol{w}_{n}|\boldsymbol{x}_{n},s_{n},\hat{\boldsymbol{\beta}})\|p(\boldsymbol{w}_{n}|\hat{\boldsymbol{\beta}}) \right) \right] \\ \#-\text{KL}\left( q_{\boldsymbol{\phi}}(\boldsymbol{c}_{n}|\boldsymbol{x}_{n},s_{n})\|p(\boldsymbol{c}_{n}) \right)-\text{KL}\left( q_{\boldsymbol{\phi}}(\boldsymbol{b}_{n}|\boldsymbol{x}_{n},s_{n})\|p(\boldsymbol{b}_{n}) \right) \end{aligned} \right]$$

$$-\sum_{k=1}^{\infty} \left[ \mathrm{KL} \left( q_{\boldsymbol{\phi}}(\hat{\beta}_{k}|\boldsymbol{X},\boldsymbol{s})\|p(\hat{\beta}_{k}) \right)+\mathrm{KL} \left( q_{\boldsymbol{\phi}}(\boldsymbol{a}_{k}|\boldsymbol{X},\boldsymbol{s})\|p(\boldsymbol{a}_{k}) \right) \right]$$

(15)

where $\mathcal{Z=\{}\hat{\boldsymbol{\beta}},\boldsymbol{W},\boldsymbol{C},\boldsymbol{B},\boldsymbol{A}\}$ represents the set of latent variables, and $q_{\boldsymbol{\phi}}\mathcal{(Z|}\boldsymbol{X},\boldsymbol{s})$ is the variational approximations of the true posteriors $p(\mathcal{Z)}$.
