# Supplementary material for: DBP: Adaptive and Interpretable Factor Analysis for Single-cell RNA-seq Data with Deep Beta Processes
Source: Genomics Proteomics Bioinformatics. 2025 Nov 26;24(3):qzaf117. doi: 10.1093/gpbjnl/qzaf117 (PMC13686767; doi:10.1093/gpbjnl/qzaf117)
Supplement: Supplementary file 1 [file qzaf117_supplementary_data.zip › qzaf117_Supplementary_data-new.zip!/Supplementary material captions.docx]

**Supplementary material**

**File S1 Derivation of the generative process**

**File S2 Derivation of the ELBO**

**Figure S1 DBP probabilistic graphical model**

**A.** DBP probabilistic graphical model for generation process. **B.** DBP probabilistic graphical model for inference process. Shaded nodes represent observed variables. Unshaded nodes represent latent variables. Arrows represent cause-effect relationships. Solid arrows define the generative process, while dashed arrows define the inferential process. Rectangles represent independent replication. DBP, deep Beta process.

**Figure S2 DBP ablation model framework**

**A.** DBP_adap model framework. **B.** DBP_naive model framework.

**Figure S3 Evaluation of DBP performance on dimensionality reduction**

**A.** UMAP visualization of the sparse factor matrix obtained by DBP and the cell embeddings obtained by eight other methods in the PBMC dataset. **B.** Benchmarking the dimensionality reduction performance of DBP and other methods on the PBMC datasets. **C.** Comparison of the runtime (left) and memory (right) costs of different methods. Gray dashed lines indicate deep learning-based methods. **D.** Overall scores of DBP under different settings of the hyperparameter $\alpha$. The red border corresponds to the default value. UMAP, Uniform Manifold Approximation and Projection; PBMC, peripheral blood mononuclear cell.

**Figure S4 Evaluation of DBP performance on biological interpretability**

**A.** PBC visualization of the sparse factor matrix obtained by DBP and the cell embedding obtained by four other methods in the lung dataset. **B.** UMAP visualization of the sparse factor values obtained by DBP and the cell embedding values obtained by other methods corresponding to CD8 T, DC, Mono, and NK cells in the PBMC dataset. PBC, polar bubble chart.

**Figure S5 Interpretable factors identified by DBP**

**A.** Violin plot visualization of the specific sparse factor values representing cell subtypes in the PBMC dataset. **B.** Violin plot visualization of the specific sparse factor values representing cell subtypes in the lung dataset. **C**. Dot plot of feature expression of the top 20 genes in specific sparse factors in the lung dataset. They ranked from top to bottom as F31, F35, and F37. **D.** GSEA results of the factor loading vector for PBMC–F22. **E.** Lollipop plot visualization of the factor loading values for the top 20 genes in the PBMC–F22. GSEA, gene set enrichment analysis.

**Figure S6 Application of DBP on GA dataset**

**A.** UMAP visualization of GA dataset. **B.** UMAP visualization of the sparse factor values of GA–F35 in the GA dataset. **C.** Lollipop plot visualization of the factor loading values for the top 20 genes in the GA–F35. GA, gastric adenocarcinoma.

**Table S1 Detailed parameter settings for SCSIM**

**Table S2 Detailed parameter settings for Splatter**

**Table S3 Hyperparameter settings for DBP training**

**Table S4 GSEA results of the PBMC dataset**

**Table S5 Genes in the PBMC dataset, sorted by factor loading matrix value**

**Table S6 GSEA results of the lung dataset**

**Table S7 Genes in the lung dataset, sorted by factor loading matrix value**

**Table S8 Genes in the GA dataset, sorted by factor loading matrix value**
